# Upper bound for broadband radiofrequency field disruption of magnetic compass orientation in night-migratory songbirds

**DOI:** 10.1073/pnas.2301153120

**Published:** 2023-07-03

**Authors:** Bo Leberecht, Siu Ying Wong, Baladev Satish, Sara Döge, Jakob Hindman, Lalitha Venkatraman, Shambhavi Apte, Katrin Haase, Isabelle Musielak, Glen Dautaj, Ilia A. Solov’yov, Michael Winklhofer, Henrik Mouritsen, P. J. Hore

**Affiliations:** ^a^Department V - School of Mathematics and Science, Institute of Biology and Environmental Sciences, Carl von Ossietzky Universität Oldenburg, Oldenburg 26129, Germany; ^b^Department V - School of Mathematics and Science, Institute of Physics, Carl von Ossietzky Universität Oldenburg, Oldenburg 26111, Germany; ^c^Research Center Neurosensory Science, University of Oldenburg, Oldenburg 26111, Germany; ^d^Department of Chemistry, Physical & Theoretical Chemistry Laboratory, University of Oxford, Oxford OX1 3QZ, United Kingdom

**Keywords:** radical pair mechanism, magnetoreception, bird migration, electrosmog, hyperfine interaction

## Abstract

Night-migratory songbirds have a light-dependent magnetic compass sense, the mechanism of which is thought to depend on the photochemical formation of radical pairs in cryptochrome (Cry) proteins located in the retina. The finding that weak radiofrequency (RF) electromagnetic fields can prevent birds from orienting in the Earth’s magnetic field has been regarded as a diagnostic test for this mechanism and as a potential source of information on the identities of the radicals. The maximum frequency that could cause such disorientation has been predicted to lie between 120 and 220 MHz for a flavin–tryptophan radical pair in Cry. Here we show that the magnetic orientation capabilities of Eurasian blackcaps (*Sylvia atricapilla*) are not affected by RF noise in the frequency bands 140 to 150 MHz and 235 to 245 MHz. From a consideration of its internal magnetic interactions, we argue that RF field effects on a flavin-containing radical-pair sensor should be approximately independent of frequency up to 116 MHz and that birds’ sensitivity to RF disorientation should fall by about two orders of magnitude when the frequency exceeds 116 MHz. Taken together with our earlier finding that 75 to 85 MHz RF fields disrupt the magnetic orientation of blackcaps, these results provide compelling evidence that the magnetic compass of migratory birds operates by a radical pair mechanism.

## Significance

Billions of small migratory songbirds fly thousands of kilometers every year between their breeding and wintering grounds, navigating with the help of an extraordinary ability to detect the direction of the Earth’s magnetic field. In behavioral tests, this ability can be disrupted by exposing birds to weak time-dependent electromagnetic fields. An important unknown quantity, which would provide vital insight into the poorly understood magnetic sensory mechanism, is the maximum frequency at which such disruption occurs. For Eurasian blackcaps, this frequency is found to lie between ~80 MHz and ~145 MHz in agreement with theoretical predictions, providing strong support for a radical-pair–based magnetic sensing mechanism.

On their biannual journeys, night-migratory songbirds navigate using a light-dependent magnetic compass (1) that responds to the inclination of the Earth’s magnetic field (2) and the wavelength of the incident light (3). Magnetic compass information, transmitted from the retina via the thalamofugal visual pathway, is processed in Cluster N, a part of the visual wulst in the birds’ forebrain (4–8). The hypothesis that chemical intermediates known as radical pairs are a crucial part of this mechanism is consistent with much of the experimental evidence, although the sensory molecule has yet to be conclusively identified (9–14).

Although weak monochromatic (single-frequency) RF (radiofrequency) fields have been reported to prevent birds from using their magnetic compass (15–20), broadband RF fields, in the frequency range from a few hundred kHz up to ~80 MHz, have been found to have a much stronger disorienting effect (20–24). While such weak, rapidly fluctuating magnetic fields should not affect a magnetite-based sensor (10, 25), their disruptive effect could be consistent with a radical pair mechanism (11, 26).

The photoreceptor protein cryptochrome (Cry) is the most likely molecule to play host to magnetically sensitive radicals. There are three known Cry genes in birds (Cry1, Cry2 and Cry4), each with two isoforms (splicing variants, a and b): Cry1a (27–30), Cry1b (28, 31, 32), Cry2a (27, 28, 33–35), Cry2b (34, 36), Cry4a (13, 36–40), and Cry4b (41). Some of them form radical pairs on light excitation (10, 13, 42–46), some are localized in photoreceptor cells within the avian retina (27, 30, 31, 39, 47), but see ref. 30, and some fulfil certain other requirements for a magnetic sensor (10, 11, 13, 27, 43, 48).

Absorption of visible light—essential for the formation of radical pairs inside Cry—is only possible if the protein binds a flavin adenine dinucleotide (FAD) chromophore. Cry4a, unlike the Cry1 and Cry2 isoforms, stoichiometrically binds FAD. Additionally, in contrast to the other five forms, Cry4a expression is seasonal rather than circadian, showing increases during the migratory season (41, 49). Cry4a therefore seems the most likely magnetic sensor (13, 36, 37, 44, 50–52). Cry4a from the European robin (*Erithacus rubecula*, *Er*), a night-migratory songbird, has recently been shown to be magnetically sensitive in vitro (13). Photoexcitation of FAD in *Er*Cry4a triggers a series of electron transfers along a chain of four tryptophans (the Trp-tetrad) to the FAD, resulting in a magnetically responsive radical pair, [FAD^•−^ TrpH^•+^], comprising flavin and tryptophan radicals (13, 44). However, it is possible that different magnetically sensitive states of Cry could be formed in vivo (16, 47, 53–56). In particular, hypothetical radical pairs in which the radical that partners FAD^•−^ has no or very few ^1^H and ^14^N hyperfine interactions could show larger magnetic field effects than [FAD^•−^ TrpH^•+^] (57).

An indication of the likely disruptive effect of time-dependent magnetic fields on the performance of a radical pair sensor may be gained from its “action-spectrum histogram,” a concept proposed by Hiscock et al. (26) and developed qualitatively by Leberecht et al. (24). Such calculations suggest that the resonant response of a radical pair to an RF field should be fairly independent of frequency up to a maximum, or “cutoff,” frequency above which the RF field should have no effect. In the case of [ ${\mathrm{FAD}}^{∙-}$ ${\mathrm{TrpH}}^{∙+}$ ], this frequency, whose value is determined principally by the hyperfine interactions in the two radicals, was predicted to lie between ~120 MHz and ~220 MHz (26). For ${\mathrm{FAD}}^{∙-}$ -containing pairs in which the partner radical has fewer and/or smaller hyperfine interactions than ${\mathrm{TrpH}}^{∙+}$ , the upper limit of this range should be reduced (24). Experimental determination of the cutoff frequency should therefore provide insight into the properties and identities of the sensory radicals. If the measured frequency were consistent with a detailed theoretical prediction, it would also provide a powerful argument against a sensory mechanism based on completely different principles (e.g., magnetic nanoparticles, reviewed in ref. 58) and against RF-induced disorientation being an experimental artefact. The 120 to 220 MHz prediction suggests three distinct behavioural tests of the Cry hypothesis: 1) a positive control using an RF field oscillating at a frequency not far below 120 MHz; 2) a negative control at a frequency above 220 MHz; and 3) measurements at frequencies within the 120 to 220 MHz range aimed at determining the cutoff frequency. Experiment (1) was the subject of Leberecht et al. (24) who found that night-migratory Eurasian blackcaps (*Sylvia atricapilla*) are disoriented by 75 to 85 MHz RF noise as expected from the predicted cutoff frequency (>~120 MHz). Experiments (2) and (3) are the subject of this report.

The aim of the present study is to test whether a night-migratory songbird species is disturbed by 1) RF noise at a frequency (240 ± 5 MHz) just above the maximum theoretically predicted cutoff and 2) by RF noise at a frequency (145 ± 5 MHz) just above the minimum theoretically predicted cutoff and 3) to present calculations of action-spectrum histograms that clarify the influence of electron–electron dipolar coupling and hence define much more precisely the likely cutoff frequency for a [ ${\mathrm{FAD}}^{∙-}$ ${\mathrm{TrpH}}^{∙+}$ ] radical pair.

## Results

### Behavioural Tests.

Based on the known magnetic properties of ${\mathrm{FAD}}^{∙-}$ and ${\mathrm{TrpH}}^{∙+}$ radicals, Hiscock et al. proposed that RF fields at frequencies above ~220 MHz should have no effect on the ability of migratory birds to orient in the Earth’s magnetic field (26). We tested this prediction by exposing Eurasian blackcaps during the 2020 and 2021 autumn migratory seasons to broadband, noise-modulated RF fields in the frequency range 235 to 245 MHz with strengths similar to the 75 to 85 MHz fields that had previously been found to disorient birds of the same species (24). If disorientation were found to occur at 235 to 245 MHz, it would cast doubt on the hypothesis that the magnetically sensitive radical pair formed in vivo is the same as in purified *Er*Cry4a in vitro, i.e. [ ${\mathrm{FAD}}^{∙-}$ ${\mathrm{TrpH}}^{∙+}$ ] (13). An experimentally determined cutoff frequency above ~220 MHz would instead be consistent with a radical pair that had substantially larger hyperfine interactions than ${\mathrm{FAD}}^{∙-}$ and/or ${\mathrm{TrpH}}^{∙+}$ . During the following spring (2022), we performed tests using 140 to 150 MHz RF fields aimed at determining whether the cutoff frequency lies between ~80 MHz and ~145 MHz.

**Fig. 1.**
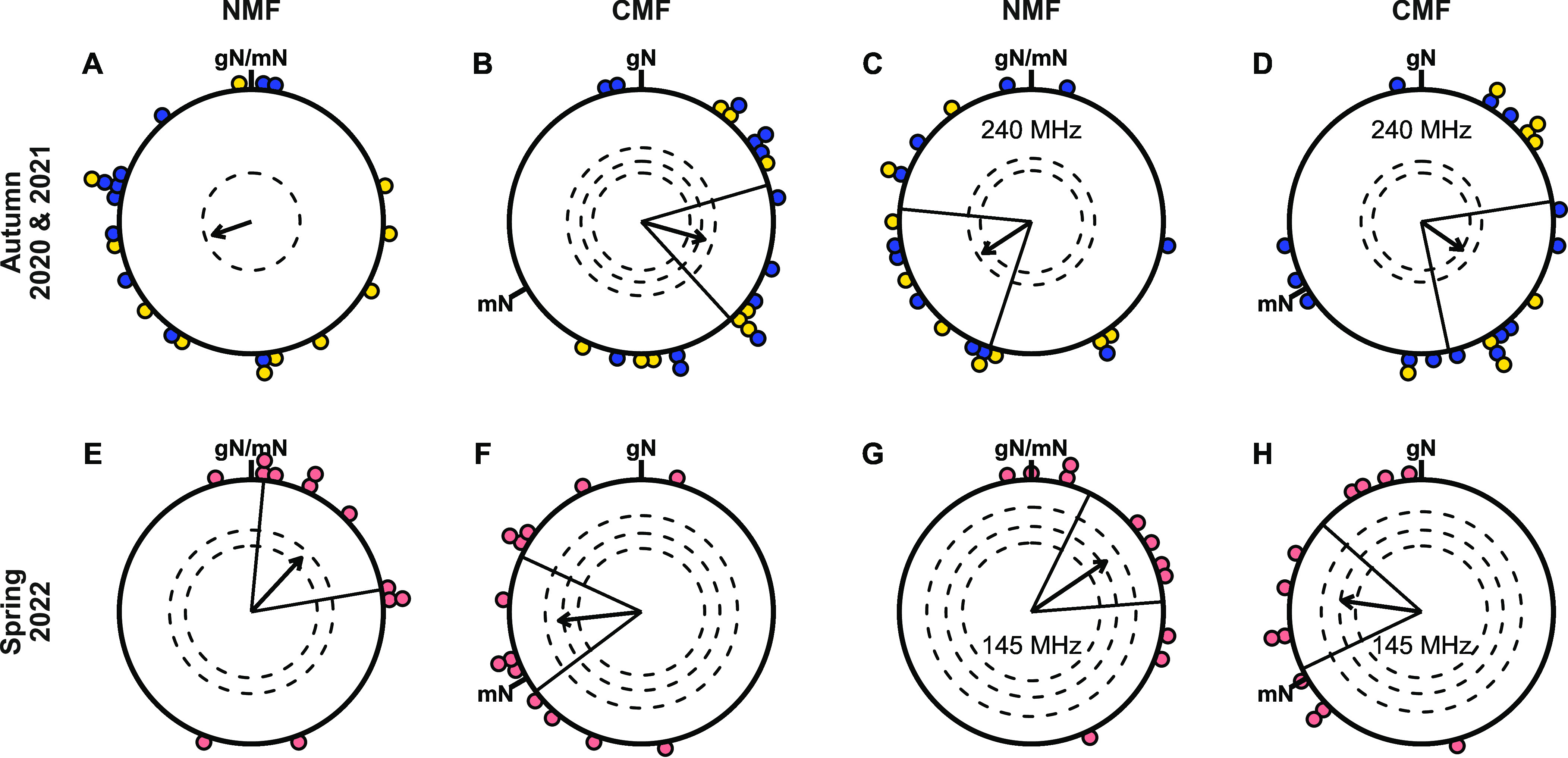
Magnetic compass orientation of Eurasian blackcaps. (*A*–*D*) Autumn migratory season of 2020 and 2021; (*E*–*H*) Spring migratory season of 2022; (*A* and *E*) NMF: normal Earth’s magnetic field in Oldenburg (*A*: N = 22; *E*: N = 12); (*B* and *F*) CMF: 120°-counter-clockwise rotated magnetic field (*B*: N = 22; *F*: N = 13); (*C*) NMF-240: NMF with a 235 to 245 MHz RF field (N = 20); (*D*) CMF-240: CMF with a 235 to 245 MHz RF field (N = 22); (*G*) NMF-145: NMF with a 140 to 150 MHz RF field (N = 11); (*H*) CMF-145: CMF with a 140 to 150 MHz RF field (N = 12). Each coloured dot represents the mean direction of one individual bird rounded to the nearest 5°. The arrows display the mean orientations of all birds tested in each condition, framed by the 95% CIs as solid lines (only present in significantly oriented groups). The arrow lengths represent the directedness of the groups in the form of their Rayleigh values (r value). Dashed circles indicate threshold *P* levels (from inner to outer circle: 0.05, 0.01, 0.001) of the Rayleigh test for the corresponding sample size; an arrow crossing a dashed circle indicates the level of significant orientation. gN: geographical North; mN: magnetic North. Yellow dots: birds in the autumn 2020 cohort; blue dots: birds in the autumn 2021 cohort; red dots: birds in the spring 2022 cohort. The same individuals were tested in all four conditions of the respective migratory season. In some cases, individual birds did not provide enough active and directed tests in all conditions with the result that the four sample sizes are not all identical.

In the normal geomagnetic field conditions in Oldenburg (NMF) during the autumn migration season, a control group of birds tested without applying RF fields tended, on average, to orient in a southwesterly direction, similar to the migratory direction of free-flying blackcaps in autumn (Fig. 1*A*: *N* = 22, mean ± SD = 251° ± 88°, *r* = 0.31, *P* = 0.1228). The lack of significance in the NMF control condition would have been critical if the RF-exposed birds had been disoriented, but they were not (see Fig. 1 *C* and *D* and below). When the magnetic field was turned 120° counter-clockwise in the horizontal plane (CMF), the birds rotated their mean orientation accordingly, now heading significantly towards east-southeast (Fig. 1*B*: *N* = 22, mean ± SD = 106° ± 68°, *r* = 0.50, *P* = 0.0034, 95% CI = ±31.9°). In the following spring, the control group of blackcaps in the NMF condition oriented in a north-easterly direction, comparable to the spring direction of their free-flying conspecifics (Fig. 1*E*: *N* = 12, mean ± SD = 43° ± 62°, *r* = 0.56, *P* = 0.0205, CI = ±37.5°). In the CMF condition, the group of birds adjusted their bearing with the turned magnetic field, now heading west (Fig. 1*F*: *N* = 13; mean ± SD = 264° ± 55°; *r* = 0.63; *P* = 0.004; CI = ±30.8°).

The group of birds exposed to 235 to 245 MHz broadband RF fields at an intensity of 3.601 $\mathrm{pT}/\sqrt{\mathrm{Hz}}$ (maxhold mode; *SI Appendix*, Table S8; labeled “240” in the following) oriented significantly in the expected autumn migratory direction in the NMF condition (Fig. 1*C*, NMF-240: *N* = 20, mean ± SD = 237° ± 74°, *r* = 0.44, *P* = 0.0201, CI = ±38.7°) and rotated their heading direction in the CMF condition (Fig. 1*D*, CMF-240: *N* = 22, mean ± SD = 127° ± 78°, *r* = 0.40, *P* = 0.0275, CI = ±40.5°). The group of blackcaps exposed to 140 to 150 MHz broadband RF fields of 2.813 $\mathrm{pT}/\sqrt{\mathrm{Hz}}$ intensity (maxhold mode; *SI Appendix*, Table S8; labelled “145” in the following) also oriented significantly in their expected spring migratory direction in the NMF condition (Fig. 1*G*, NMF-145: *N* = 11, mean ± SD = 56° ± 50°, *r* = 0.68, *P* = 0.0036, CI = ±29.7°), and changed their heading in the CMF condition accordingly (Fig. 1*H*, CMF-145: *N* = 12, mean ± SD = 278° ± 57°, *r* = 0.61, *P* = 0.009, CI = ±33.6°).

The birds’ headings differed significantly between the NMF condition and the 120°-counter-clockwise rotated CMF condition in both the presence and absence of the applied RF fields (Mardia–Watson–Wheeler test: Autumn: NMF–CMF: *ω*_(degrees of freedom = 2)_ = 10.174, *P* = 0.0062; NMF-240–CMF-240: *ω*_(2__)_ = 8.805, *P* = 0.0123; Spring: NMF–CMF: *ω*_(2__)_ = 14.448, *P* = 0.0007; NMF-145–CMF-145: *ω*_(2__)_ = 16.592, *P* = 0.0003). The headings in all CMF conditions with and without RF fields present were significantly distributed around their corresponding NMF mean angles rotated counterclockwise by 120° (V-Test: Autumn: CMF against 131°: *V* = 0.450, *µ* = 2.98, *P* = 0.0012; CMF-240 against 117°: *V* = 0.394, *µ* = 2.61, *P* = 0.0041; Spring: CMF against 283°: *V* = 0.594, *µ* = 3.03, *P* = 0.0009; CMF-145 against 296°: *V* = 0.578, *µ* = 2.83, *P* = 0.0018). In neither condition (NMF or CMF in either migratory season) was there a significant difference between the data with and without the RF fields (Mardia-Watson-Wheeler test: Autumn: NMF–NMF-240: *ω*_(2__)_ = 0.383, *P* = 0.8259; CMF–CMF-240: *ω*_(2__)_ = 1.009, *P* = 0.6038; Spring: NMF–NMF-145: *ω*_(2__)_ = 0.547, *P* = 0.7606; CMF–CMF-145: *ω*_(2__)_ = 0.197, *P* = 0.9061).

Bootstrapping, with 100,000 iterations (19, 24, 59), was used to test whether the orientation results obtained in the NMF condition without the 235 to 245 MHz RF field (NMF, Fig. 1*A*) were significantly more random than the corresponding results with the RF field present (NMF-240, Fig. 1*C*). The bootstrap for the NMF condition showed that 73.8% (*P* = 0.7383) of the iterations reached or surpassed the directedness (*r* = 0.44) of the significantly oriented NMF-240 condition. Of these, 78.6% (*P* = 0.7859) also lay within the CIs (198.2° to 275.6°) of the NMF-240 condition. Hence, 58.0% of the bootstrap iterations were as directed and oriented as the RF field counterpart. The orientation results of the NMF control condition are therefore most likely not of random nature and would be expected eventually to become significantly oriented with a higher sample size (see discussion in *SI Appendix*, section S4.5).

To summarize, the magnetic orientation ability of the Eurasian blackcaps tested in this study was not affected by 235 to 245 MHz RF fields or by 140 to 150 MHz RF fields at intensities (3.601 $\mathrm{pT}/\sqrt{\mathrm{Hz}}$ and 2.813 $\mathrm{pT}/\sqrt{\mathrm{Hz}}$ , respectively; *SI Appendix*, Table S8) comparable to those that have previously been found to cause disorientation at 75 to 85 MHz.

### Action-Spectrum Histograms.

The action-spectrum histogram of a radical pair is obtained from the eigenvalue spectrum of the spin Hamiltonian in the absence of an RF field by first identifying energy levels whose separation falls within a certain frequency interval (26). For each pair of energy levels that satisfy this condition, the probability that an RF field would induce a transition between them is multiplied by the difference in their populations at the moment the radical pair is formed in a singlet state. The resulting quantities, termed “resonance effects,” are summed for each frequency interval, averaged over a uniform distribution of magnetic field directions, and presented as a histogram of average resonance effect against frequency.

**Fig. 2.**
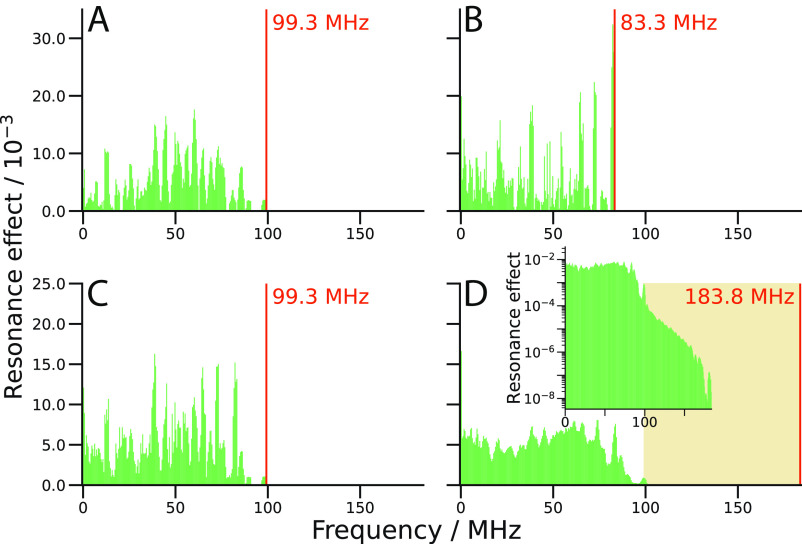
Action-spectrum histograms of (*A*) [ ${\mathrm{FAD}}^{∙-}$ $Z^{∙}$ ], (*B*) [ ${\mathrm{TrpH}}^{∙+}$ $Z^{∙}$ ], and (*C* and *D*) [ ${\mathrm{FAD}}^{∙-}$ ${\mathrm{TrpH}}^{∙+}$ ] in the absence (*A*, *B*, and *C*) and presence (*D*) of dipolar coupling (*D* = −14.3 MHz). The number of nuclei included in ${\mathrm{FAD}}^{∙-}$ and ${\mathrm{TrpH}}^{∙+}$ are 7 and 4, respectively (*SI Appendix*, section S1) and the geomagnetic field strength was 50 μT. The vertical orange lines indicate the cutoff frequency in each plot. The *Inset* in (*D*) is the same histogram plotted on a logarithmic vertical scale. In both (*D*) and its *Inset*, the region above 99.3 MHz [i.e., the cutoff frequency for the uncoupled (*D* = 0) radical pair] is shaded in pale orange. In (*A*) and (*B*), the strong, narrow contribution from the $Z^{∙}$ radical (at the electron Larmor frequency, 1.4 MHz) has been omitted. The histogram bins are 0.5 MHz wide.

The spin system of [ ${\mathrm{FAD}}^{∙-}$ ${\mathrm{TrpH}}^{∙+}$ ] (two dipolar-coupled electrons and a total of 27 hyperfine-coupled nuclei, 15 in ${\mathrm{FAD}}^{∙-}$ and 12 in ${\mathrm{TrpH}}^{∙+}$ , *SI Appendix*, section S1) is too large to allow its action-spectrum histogram to be calculated (roughly estimated to require random-access memory on the order of exabytes). To explore the general properties of these histograms, we therefore started with a radical pair containing a reduced set of nuclear spins comprising the nitrogen and hydrogen atoms with the seven largest hyperfine interactions in ${\mathrm{FAD}}^{∙-}$ and the four largest in ${\mathrm{TrpH}}^{∙+}$ . Fig. 2*C* shows the histogram for this truncated “uncoupled” spin system in which the dipolar interaction of the two radicals, *D*, was set equal to zero. It contains a “forest” of resonances covering the range of RF frequencies up to, but not beyond 99.3 MHz.

Insight into the contributions of the individual radicals to Fig. 2*C* can be obtained from the histograms for the simpler, uncoupled radical pairs [ ${\mathrm{FAD}}^{∙-}$ $Z^{∙}$ ] and [ ${\mathrm{TrpH}}^{∙+}$ $Z^{∙}$ ] shown in Fig. 2 *A* and *B*, respectively, where $Z^{∙}$ is a radical with no hyperfine interactions whose only contribution to the histograms would be at 1.4 MHz, the Larmor frequency of a free electron in the 50 μT geomagnetic field (11). [ ${\mathrm{FAD}}^{∙-}$ $Z^{∙}$ ] and [ ${\mathrm{TrpH}}^{∙+}$ $Z^{∙}$ ] have clear cutoff frequencies, at 99.3 MHz and 83.3 MHz, respectively. The histogram for [ ${\mathrm{FAD}}^{∙-}$ ${\mathrm{TrpH}}^{∙+}$ ] resembles the sum of Fig. 2 *A* and *B*, with a cutoff frequency equal to the larger of the values for [ ${\mathrm{FAD}}^{∙-}$ $Z^{∙}$ ] and [ ${\mathrm{TrpH}}^{∙+}$ $Z^{∙}$ ], i.e., 99.3 MHz. There are no resonance effects above 99.3 MHz in Fig. 2*C* because RF-induced transitions involving both radicals are forbidden when there is no spin–spin coupling, i.e., when *D* = 0 (*SI Appendix*, section S3).

The histogram for this truncated model of [ ${\mathrm{FAD}}^{∙-}$ ${\mathrm{TrpH}}^{∙+}$ ] (Fig. 2*C*) is not greatly changed when a realistic dipolar interaction [*D* = −14.3 MHz, appropriate for the 1.76 nm distance between ${\mathrm{FAD}}^{∙-}$ and the penultimate tryptophan radical of the Trp-tetrad in avian Cry4a (44)] is introduced (Fig. 2*D*). The forest of histogram bars for this more realistic, coupled radical pair is smoother (less “spiky”), reflecting the larger number of distinct transitions. Weak resonances appear at frequencies above 99.3 MHz, composed of a) previously allowed transitions that have been shifted to higher frequencies by the dipolar interaction and b) previously forbidden transitions, involving both electrons, that have become partially allowed (*SI Appendix*, section S3). The former dominates and occurs exclusively in the first ~10 MHz above 99.3 MHz. The latter appear at frequencies up to a new cutoff of 183.8 MHz, very close to the sum of the cutoff frequencies in Fig. 2 *A* and *B* (99.3 + 83.3 = 182.6 MHz). This can be seen from the inset in Fig. 2*D*, where the histogram is plotted semi-logarithmically. This figure also shows that the resonances above 99.3 MHz are much weaker than those below 99.3 MHz. The sum of the heights of the histogram bars for frequencies greater than 99.3 MHz is 0.0044. In other words, RF effects at frequencies above 99.3 MHz account for less than 1% of the total effects of RF fields on this truncated spin system.

To summarize, although RF effects for [ ${\mathrm{FAD}}^{∙-}$ ${\mathrm{TrpH}}^{∙+}$ ] with *D* ≠ 0 can be expected at frequencies up to the *sum* of the cutoff frequencies for [ ${\mathrm{FAD}}^{∙-}$ $Z^{∙}$ ] and [ ${\mathrm{TrpH}}^{∙+}$ $Z^{∙}$ ], the overwhelming majority of those effects occur at frequencies *lower* than the larger of those two frequencies. There seems to be no reason why this conclusion should not apply to the complete spin system with 27 nuclei (instead of 11) and the same dipolar coupling.

### Cutoff Frequencies.

We now proceed to estimate the cutoff frequency for the complete 27-nucleus spin system of [ ${\mathrm{FAD}}^{∙-}$ ${\mathrm{TrpH}}^{∙+}$ ]. To do so, we consider $\nu_{\max}$ , defined as the frequency gap between the highest and lowest energy levels of a radical. On the basis that there can be no resonance effect at a frequency higher than this, $\nu_{\max}$ values for ${\mathrm{FAD}}^{∙-}$ and ${\mathrm{TrpH}}^{∙+}$ are expected to provide reliable estimates of the corresponding cutoff frequencies of the uncoupled [ ${\mathrm{FAD}}^{∙-}$ $Z^{∙}$ ] and [ ${\mathrm{TrpH}}^{∙+}$ $Z^{∙}$ ] radical pairs. This is certainly the case for the simplified radical pairs in Fig. 2. The values of $\nu_{\max}$ (obtained by diagonalising the spin Hamiltonians) are 99.4 and 83.5 MHz for ${\mathrm{FAD}}^{∙-}$ and ${\mathrm{TrpH}}^{∙+}$ , respectively, while the histogram cutoff frequencies (in Fig. 2 *A* and *B*) are 99.3 and 83.3 MHz. The small mis-matches arise because the cutoffs in Fig. 2 are given as the midpoints of the appropriate histogram bins.

**Fig. 3.**
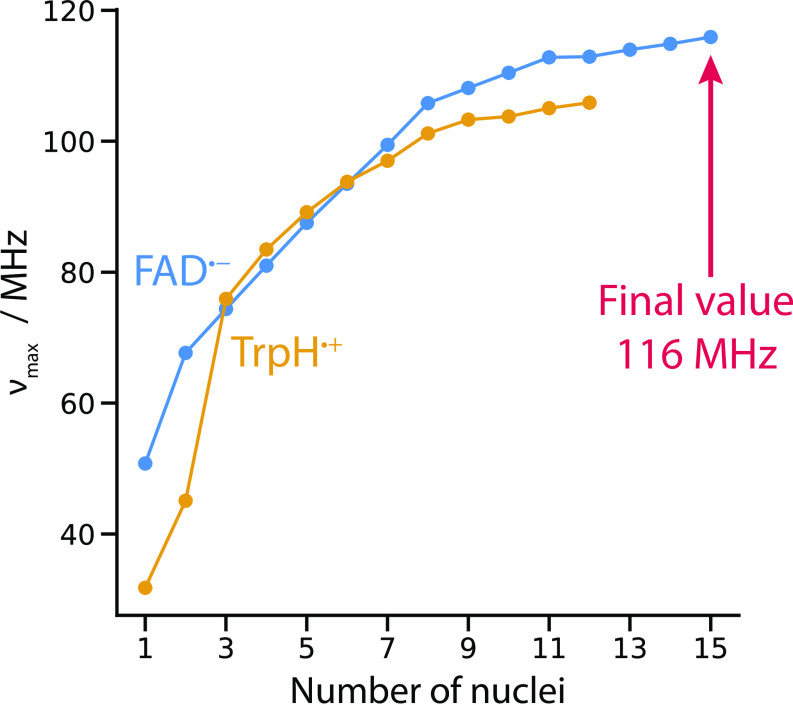
Maximum resonance frequencies, $\nu_{\max}$ for ${\mathrm{FAD}}^{∙-}$ and ${\mathrm{TrpH}}^{∙+}$ radical pairs as a function of the number of nuclei included in the calculation.

Fig. 3 shows how $\nu_{\max}$ for ${\mathrm{FAD}}^{∙-}$ and ${\mathrm{TrpH}}^{∙+}$ builds up, adding one nucleus at a time in approximate order of decreasing hyperfine interaction. It is clear that ${\mathrm{FAD}}^{∙-}$ dominates once all nuclei have been included. The final values of $\nu_{\max}$ , for ${\mathrm{FAD}}^{∙-}$ with its full complement of 15 nuclei and ${\mathrm{TrpH}}^{∙+}$ with 12 nuclei, are 116 MHz and 106 MHz, respectively. We therefore expect the cutoff frequency for [ ${\mathrm{FAD}}^{∙-}$ ${\mathrm{TrpH}}^{∙+}$ ] to be 116 MHz.

In summary, without exactly determining the cutoff frequency for the coupled 27-nuclear-spin [ ${\mathrm{FAD}}^{∙-}$ ${\mathrm{TrpH}}^{∙+}$ ] radical pair, we can be confident that there will only be very small (< 1%) RF effects at frequencies above 116 MHz, i.e., the value of $\nu_{\max}$ for ${\mathrm{FAD}}^{∙-}$.

## Discussion

**Fig. 4.**
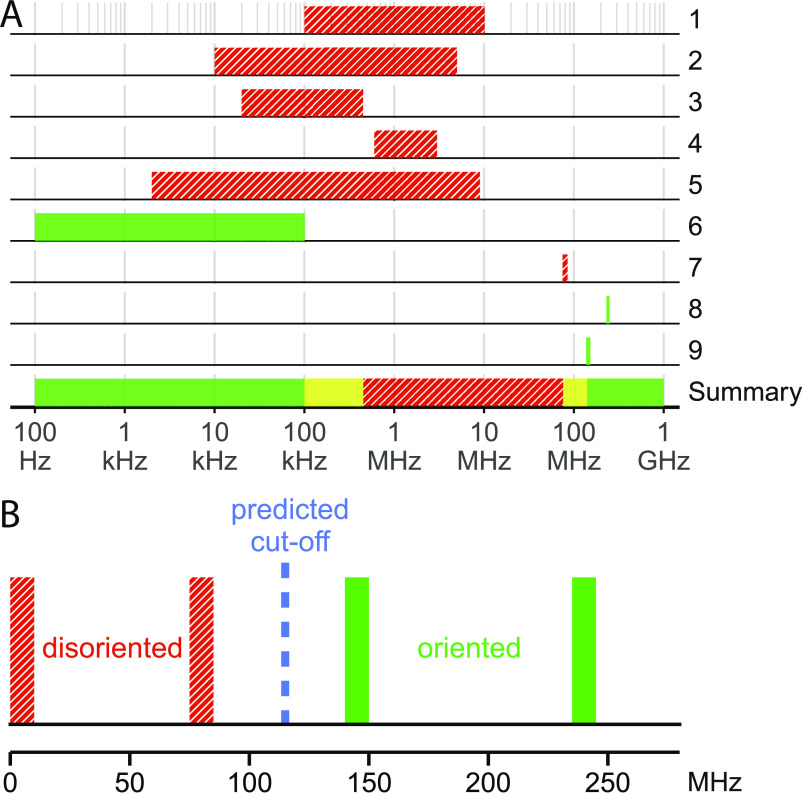
(*A* and *B*) Summary of the reported effects of broadband RF fields on the orientation behaviour of night-migratory songbirds. (*A*) Three assumptions were made: 1) the RF effects are not species-specific (data from European robin and Eurasian blackcap are merged); 2) the differences in RF field intensity between studies are negligible (their magnitudes are compared in *SI Appendix,* Table S8; 3) based on Fig. 2*D*, it is unlikely that there are sensitivity “holes” in the action spectrum histograms of the radicals. Boxes shaded in red (with crosshatching) indicate that a disruptive effect was reported; green boxes (without crosshatching) mean no disruptive effect. The lowest and highest frequencies that cause disorientation are expected to lie within the yellow boxes in the bottom row of (*A*). The green box representing the present study has been increased in width to improve visibility. (*B*) An expanded section of (*A*). Based on these data and the above assumptions, RF fields cease to have a disrupting effect on the magnetic compass orientation of night-migratory songbirds at a frequency between ~80 MHz and ~145 MHz. The following nine studies of broadband RF field effects are included in (*A*): 1) Ritz et al. (20); 2) Engels et al. (21) (Fig. 4*F*, red trace); 3) Engels et al. (21) (Fig. 4*F*, green trace); 4) Engels et al. (21) (Fig. 4*F*, black trace); 5) Schwarze et al. (22) (Fig. 3*A*, yellow trace); 6) Kobylkov et al. (23) (Fig. 1*A*); 7) Leberecht et al. (24) (Fig. 2*B*); 8) 235 to 245 MHz condition of present study (*SI Appendix*, Fig. S6*B*); 9) 140 to 150 MHz condition of present study (*SI Appendix*, Fig. S6*D*).

Calculation of action-spectrum histograms and $\nu_{\max}$ frequencies (Figs. 2 and 3) leads to the conclusion that the effective cutoff frequency for a [ ${\mathrm{FAD}}^{∙-}$ ${\mathrm{TrpH}}^{∙+}$ ] radical pair in Cry should be close to 116 MHz. The magnetic orientation ability of the Eurasian blackcaps tested in this study was not affected by 140 to 150 or 235 to 245 MHz RF fields under conditions essentially identical to earlier experiments in which 75 to 85 MHz RF fields had been found to cause disorientation (24). This finding is consistent with the predicted ~116 MHz upper limit on the frequencies capable of interfering with the spin dynamics of a [ ${\mathrm{FAD}}^{∙-}$ ${\mathrm{TrpH}}^{∙+}$ ] radical pair. Put together with previous studies of migratory songbirds exposed to broadband RF fields (20–24), the range of frequencies that interfere with birds’ ability to orient magnetically is now known to begin above 100 kHz and to end below ~145 MHz (Fig. 4*A*). The upper limit for RF disorientation now lies between ~80 MHz (24) and ~145 MHz (this study). This frequency range includes the 116 MHz predicted cutoff.

In principle, the uncertainty in the cutoff frequency could be further reduced by conducting behavioural experiments using RF fields with frequency bands f±5MHz where, for example, *f* = 90, 100, 110, … MHz. However, acquisition of sufficient data at each new frequency band would typically require tests spanning two whole migratory seasons in all three of our magnetically shielded chambers (*Methods* and *SI Appendix*, section S4) to make a confident claim of any disruptive effect at a given frequency.

The expected cutoff frequency is determined by the radical with the larger $\nu_{\max}$ (i.e., the one with the stronger hyperfine interactions) and hence our findings do not distinguish between [ ${\mathrm{FAD}}^{∙-}$ ${\mathrm{TrpH}}^{∙+}$ ] and a pair in which ${\mathrm{TrpH}}^{∙+}$ has been replaced by a radical with smaller and/or fewer hyperfine interactions. To make that distinction, as explained previously (24), it would be necessary to determine the threshold RF *intensity* required for disorientation at different frequencies within the 1 to 80 MHz range, a challenge even more daunting than testing for orientation/disorientation at multiple frequenices between ~80 and ~145 MHz.

Our experiments, together with detailed theoretical predictions, provide strong evidence that the compass magnetoreceptor in migratory birds is based on a flavin-containing radical pair and not a completely different sort of receptor, for example one based on magnetic nanoparticles (*SI Appendix*, section S6). Even if plausible reasons existed for thinking that such particles could respond to weak RF fields, it would be an extraordinary coincidence if those effects had a sharp cutoff frequency within ~30 MHz of 116 MHz, as confidently predicted for a flavin-containing radical pair. The same logic also argues strongly against RF-disorientation being a bizarre effect on the bird’s motivation to orient or some form of interference with another aspect of magnetoreception, e.g., signal transduction (26).

## Methods

### Behavioural Experiments.

The procedures for the behavioural tests were essentially identical to those of Leberecht et al. (24): Full details are given there and in *SI Appendix*, section S4. The only differences were in the choice of the RF frequency band (140 to 150 MHz or 235 to 245 MHz instead of 75 to 85 MHz) and the timing of the experiments. Leberecht et al. (24) tested Eurasian blackcaps in the spring migratory season of 2019 and 2021. The experiments reported here were conducted in the autumn migratory seasons of 2020 and 2021 (235 to 245 MHz) and the spring of 2022 (140 to 150 MHz) using birds that had been wild-caught after the breeding season, during the autumn migration, in the immediate vicinity of the University of Oldenburg. Birds tested in the autumn of 2020 were not used in the tests in the autumn of 2021, while a couple of birds from both autumn cohorts were used in spring 2022. Due to the clear results of the experiments in spring 2022, it was not necessary to repeat the measurements at 140 to 150 MHz in a second migratory season.

### Spin Dynamics Calculations.

RF action-spectrum histograms were calculated as described by Hiscock et al. (26). The height of the histogram bar centred at frequency ν , covering the interval $\left[\nu -\frac{1}{2}\Delta \nu ,\;\nu +\frac{1}{2}\Delta \nu \right]$ , is given by

[1] $$\sum_{\nu_{\mathrm{ij}}\;\in \;\left[\nu -\frac{1}{2}\Delta \nu ,\;\nu +\frac{1}{2}\Delta \nu \right]}N{\left|\langle i|{\hat{H}}_{⊥}|j\rangle \right|}^{2}\left|\langle i|{\hat{P}}_{S}|i\rangle -\langle j|{\hat{P}}_{S}|j\rangle \right|,$$

where $|i\rangle$ and $|j\rangle$ are eigenstates of the spin Hamiltonian ${\hat{H}}_{0}$ (containing the geomagnetic Zeeman, hyperfine and dipolar interactions, *SI Appendix*, section S1) which are separated in energy by $h\nu_{\mathrm{ij}}=\left|\langle i|{\hat{H}}_{0}|i\rangle -\langle j|{\hat{H}}_{0}|j\rangle \right|$ . ${\hat{P}}_{S}$ is the singlet projection operator, ${\hat{H}}_{⊥}$ is proportional to the Zeeman Hamiltonian for a static field perpendicular to the geomagnetic field, and *N* scales the heights of the bars so that they sum to unity. The two parts of Eq. (**1**) are proportional, respectively, to the probability that a transition between $|i\rangle$ and $|j\rangle$ is induced by an RF field and the difference in the populations of these two states when the radical pair is formed in a singlet state. The width of the histogram bins, Δν , was 0.5 MHz and the spectra were averaged over 199 randomly distributed directions of the geomagnetic field (50 μT).

The hyperfine and dipolar coupling tensors (*SI Appendix*, section S1) were rotated to match the relative orientation of FAD and Trp_C_H (the third tryptophan of the Trp-tetrad) in the X-ray structure of pigeon (*Columba livia, Cl*) Cry4a (44). While the radical pair [ ${\mathrm{FAD}}^{∙-}$ ${\mathrm{Trp}}_{D}H_{}^{∙+}$ ] may contribute to the magnetic sensitivity of Cry4a, as part of a composite radical pair together with [ ${\mathrm{FAD}}^{∙-}$ ${\mathrm{Trp}}_{C}H_{}^{∙+}$ ] (13, 48), we only consider the latter i) for simplicity and ii) to have the maximum possible dipolar coupling. The center-to-center distance between FAD and Trp_C_H in *Cl*Cry4a is 1.76 nm, corresponding to a dipolar coupling *D* = −14.3 MHz. The exchange interaction of the two radicals is approximately 100 times smaller (13) and was consequently omitted.

### Ethics.

All experimental procedures were conducted in accordance with national and local guidelines for the use of animals in research, approved by the Animal Care and Use Committees of the Niedersächsisches Landesamt für Verbraucherschutz und Lebensmittelsicherheit (LAVES, Oldenburg, Germany, 33.19-42502-04-17/2724).

## Supplementary Material

Appendix 01 (PDF)

## Data Availability

Code is available at: https://gitlab.uni-oldenburg.de/quantbiolab/actionspectrumhistograms. All study data are included in the article and/or *SI Appendix*.
