# Supplementary material for: Upper bound for broadband radiofrequency field disruption of magnetic compass orientation in night-migratory songbirds
Source: Proc Natl Acad Sci U S A. 2023 Jul 3;120(28):e2301153120. doi: 10.1073/pnas.2301153120 (PMC10334787; doi:10.1073/pnas.2301153120)
Supplement: Supplementary file 1 — Appendix 01 (PDF) [file pnas.2301153120.sapp.pdf]

**Upper bound for broadband radiofrequency field disruption of magnetic compass orientation in night-migratory songbirds**

Bo Leberecht[1§], Siu Ying Wong[2§], Baladev Satish[1], Sara Döge[1], Jakob Hindman[1], Lalitha Venkatraman[1], Shambhavi Apte[1], Katrin Haase[1], Isabelle Musielak[1], Glen Dautaj[1], Ilia A. Solov'yov[2,3], Michael Winklhofer[1,3], Henrik Mouritsen[1,3]*, and P. J. Hore[4]*

[1] Institute for Biology and Environmental Sciences, Carl von Ossietzky Universität Oldenburg, Oldenburg, 26129, Germany.

[2] Institute for Physics, Carl von Ossietzky Universität Oldenburg, Oldenburg, 26111, Germany.

[3] Research Center Neurosensory Science, University of Oldenburg, Oldenburg, 26111, Germany.

[4] Department of Chemistry, University of Oxford, Oxford, OX1 3QZ, United Kingdom.

[§]These authors contributed equally

*Corresponding authors: peter.hore@chem.ox.ac.uk and henrik.mouritsen@uni-oldenburg.de

# Supplementary Information

## Table of Contents

S1. Supplementary Methods: spin dynamics calculations ..... 2

S1.1. Spin Hamiltonian ..... 2

S1.2. Spin Hamiltonian parameters ..... 2

S1.3. Direction of the radiofrequency field ..... 7

S2. Supplementary Results: [FAD$^{\bullet-}$ Z$^{\bullet}$] radical pairs ..... 8

S3. Supplementary Results: dipolar coupling as a perturbation ..... 10

S4. Supplementary Methods: behavioural experiments ..... 12

S4.1. Testing site ..... 12

S4.2. Generation and measurement of static magnetic field stimuli ..... 12

S4.3. Generation and measurement of time-dependent electromagnetic fields ..... 12

S4.4. Acquisition and analysis of behavioural data ..... 15

S4.5. Elaboration on analysis of the autumn NMF control condition ..... 17

S5. Supplementary Tables ..... 19

S6. Estimation of radiofrequency magnetic field effects on a magnetite-particle-based receptor ..... 22

S7. References ..... 23

# S1. Supplementary Methods: spin dynamics calculations

## S1.1. Spin Hamiltonian

The spin Hamiltonian, $\hat{H}_0$, used to calculate action spectrum histograms (Eq. (1)), has the form:

$$\hat{H}_0 = \boldsymbol{\omega}.(\hat{\mathbf{S}}_{\mathrm{A}} + \hat{\mathbf{S}}_{\mathrm{B}}) + \hat{\mathbf{S}}_{\mathrm{A}}.\mathbf{D}.\hat{\mathbf{S}}_{\mathrm{B}} + \sum_{i\in\{\mathrm{A,B}\}} \sum_k \hat{\mathbf{S}}_i.\mathbf{A}_{i,k}.\hat{\mathbf{I}}_{i,k} \qquad \text{(S1)}$$

where the A and B subscripts denote the electrons in each radical, $\hat{\mathbf{S}}_i = \left(\hat{S}_{ix}, \hat{S}_{iy}, \hat{S}_{iz}\right)$ is the vector of spin operators for electron *i*, $-\boldsymbol{\omega}/\gamma_{\mathrm{e}}$ is the geomagnetic field vector, **D** is the electron-electron dipolar coupling tensor, and $\mathbf{A}_{i,k}$ and $\hat{\mathbf{I}}_{i,k} = \left(\hat{I}_{i,kx}, \hat{I}_{i,ky}, \hat{I}_{i,kz}\right)$ are, respectively, the hyperfine coupling tensor and the vector of spin operators for nucleus *k* in radical *i*.

In the absence of dipolar coupling, the spin Hamiltonian in Eq. (S1) can be rewritten

$$\hat{H}_0 = \hat{H}_{\mathrm{A}} + \hat{H}_{\mathrm{B}}, \qquad \text{(S2)}$$

where

$$\hat{H}_i = \boldsymbol{\omega}\cdot\hat{\mathbf{S}}_i + \sum_k \hat{\mathbf{S}}_i.\mathbf{A}_{i,k}.\hat{\mathbf{I}}_{i,k}, \quad i\in\{\mathrm{A,B}\}, \qquad \text{(S3)}$$

i.e. as a sum of the individual radical spin Hamiltonians.

## S1.2. Spin Hamiltonian parameters

Action-spectrum histograms were computed according to Eq. (1) using Eq. (S1). The strength of the geomagnetic field $(-\boldsymbol{\omega}/\gamma_{\mathrm{e}})$ was 50 μT. The two-angle repulsion grid "rep_2ang_400pts_hem" in the *Spinach* spin dynamics library (1) was used to generate 199 directions of the geomagnetic field $\boldsymbol{\omega}$ distributed uniformly over a hemisphere. "Average" action-spectrum histograms were obtained by taking the arithmetic mean of the histograms for the 199 field directions.

We note that the histograms would be identical for a radical pair formed in a triplet state. Eq. (1) can be used for an initial triplet by replacing $\hat{P}_{\mathrm{S}}$ by $\hat{P}_{\mathrm{T}}$ (= $\hat{E} - \hat{P}_{\mathrm{S}}$, where $\hat{E}$ is the identity operator):

$$\langle i|\hat{P}_{\mathrm{T}}|i\rangle = \langle i|\hat{E}|i\rangle - \langle i|\hat{P}_{\mathrm{S}}|i\rangle = \langle i|i\rangle - \langle i|\hat{P}_{\mathrm{S}}|i\rangle = 1 - \langle i|\hat{P}_{\mathrm{S}}|i\rangle,$$

hence

$$\left|\langle i|\hat{P}_{\mathrm{T}}|i\rangle - \langle j|\hat{P}_{\mathrm{T}}|j\rangle\right| = \left|\left(1-\langle i|\hat{P}_{\mathrm{S}}|i\rangle\right) - \left(1-\langle j|\hat{P}_{\mathrm{S}}|j\rangle\right)\right| = \left|-\langle i|\hat{P}_{\mathrm{S}}|i\rangle + \langle j|\hat{P}_{\mathrm{S}}|j\rangle\right| = \left|\langle i|\hat{P}_{\mathrm{S}}|i\rangle - \langle j|\hat{P}_{\mathrm{S}}|j\rangle\right|.$$

That is, Eq. (1), and therefore the histograms, are unchanged by replacing $\hat{P}_{\mathrm{S}}$ by $\hat{P}_{\mathrm{T}}$.

The hyperfine tensors $\mathbf{A}_{i,k}$ and dipolar tensor **D** are based on the positions and relative orientations of FAD and $\mathrm{Trp_CH}$ (Trp318) in the X-ray structure of pigeon cryptochrome 4a (*Cl*Cry4a, PDB: 6PU0 (2)). The atomic labelling schemes for FAD and TrpH are given in Fig. S1A and S1B, respectively; standard PDB/IUPAC nomenclature is used.

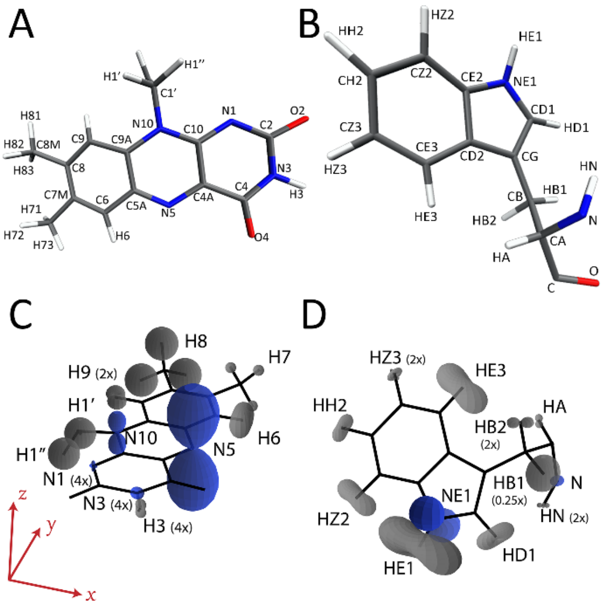


**Fig. S1**. (A) Atom labelling scheme for FAD. (B) Atom labelling scheme for TrpH. (C) Principal axis system of FAD and hyperfine tensor surface plots for $FAD^{\bullet-}$. (D) Hyperfine tensor surface plots for $TrpH^{\bullet+}$ (3).

The [$FAD^{\bullet-}$ $TrpH^{\bullet+}$] radical pair contains 15 hyperfine-coupled nuclei in $FAD^{\bullet-}$ and 12 in $TrpH^{\bullet+}$. The hyperfine tensors were calculated using density functional theory and rotated to the principal axis system of the FAD, in which the *z*-axis is perpendicular to the plane of the flavin, the *x*-axis is parallel to the vector joining N5 and N10, and the *y*-axis is the long axis of the tricyclic flavin group, perpendicular to *x* and *z*. The $TrpH^{\bullet+}$ tensors were rotated to match the relative orientation of FAD and $Trp_{C}H$ in the crystal structure of *Cl*Cry4a.

Fig. S1C and S1D show surface plots of the hyperfine tensors, together with the FAD axis system (Fig. S1C). The elements of the 27 hyperfine tensors are given in Tables S1 and S2.

The dipolar tensor **D** is constructed from the vector connecting the centres of spin density in $FAD^{\bullet-}$ and $Trp_{C}H^{\bullet+}$ in the principal axis system of $FAD^{\bullet-}$, and is given in Table S3. The eigenvalues of **D** are $\{2D/3,\ 2D/3, -4D/3\}$, where the dipolar coupling strength, $D = -14.3$ MHz.

| FAD$^{•−}$ Hyperfine Coupling Tensors / MHz | | | |
|---|---|---|---|
| N5 | −2.79 | −0.08 | 0.00 |
| | −0.08 | −2.45 | 0.00 |
| | 0.00 | 0.00 | 49.24 |
| N10 | −0.42 | −0.06 | 0.00 |
| | −0.06 | −0.66 | 0.00 |
| | 0.00 | 0.00 | 16.94 |
| H8 (3 protons) | 12.33 | 0.00 | 0.00 |
| | 0.00 | 12.33 | 0.00 |
| | 0.00 | 0.00 | 12.33 |
| H1' | 11.41 | 0.00 | 0.00 |
| | 0.00 | 11.41 | 0.00 |
| | 0.00 | 0.00 | 11.41 |
| H1" | 11.41 | 0.00 | 0.00 |
| | 0.00 | 11.41 | 0.00 |
| | 0.00 | 0.00 | 11.41 |
| H6 | −5.63 | 0.92 | 0.00 |
| | 0.92 | −14.77 | 0.00 |
| | 0.00 | 0.00 | −12.15 |
| H7 (3 protons) | −3.97 | 0.00 | 0.00 |
| | 0.00 | −3.97 | 0.00 |
| | 0.00 | 0.00 | −3.97 |
| H9 | 1.88 | −0.71 | 0.00 |
| | −0.71 | 3.01 | 0.00 |
| | 0.00 | 0.00 | −0.14 |
| N3 | −1.21 | 0.00 | 0.00 |
| | 0.00 | −0.92 | 0.00 |
| | 0.00 | 0.00 | −1.09 |
| H3 | −0.84 | −0.07 | 0.00 |
| | −0.07 | 1.06 | 0.00 |
| | 0.00 | 0.00 | −1.81 |
| N1 | −0.72 | 0.08 | 0.00 |
| | 0.08 | −0.64 | 0.00 |
| | 0.00 | 0.00 | 1.07 |

**Table S1**. Elements (in MHz) of the FAD$^{•−}$ hyperfine coupling tensors, in the flavin axis system, calculated (by Dr Ilya Kuprov, Department of Chemistry, University of Southampton (4)) using density functional theory in Gaussian-03 (5) at the UB3LYP/EPR-III level for the radical anion of 7,8,10-trimethyl isoalloxazine (lumiflavin).

| TrpH$^{\bullet+}$ Hyperfine Coupling Tensors / MHz | | | |
|---|---|---|---|
| NE1 | −1.48 | 1.64 | −1.29 |
| | 1.64 | 15.82 | −15.83 |
| | −1.29 | −15.83 | 12.70 |
| HE1 | −28.05 | 5.78 | 5.42 |
| | 5.78 | −12.38 | 8.61 |
| | 5.42 | 8.61 | −9.88 |
| HB1 | 44.07 | 0.44 | 1.33 |
| | 0.44 | 42.47 | 1.77 |
| | 1.33 | 1.77 | 48.36 |
| HE3 | −16.01 | 4.51 | 5.49 |
| | 4.51 | −13.58 | 2.34 |
| | 5.49 | 2.34 | −11.44 |
| HZ2 | −12.40 | 3.56 | 4.17 |
| | 3.56 | −9.93 | 2.67 |
| | 4.17 | 2.67 | −8.25 |
| HD1 | −7.72 | −4.40 | −4.89 |
| | −4.40 | −7.66 | 2.59 |
| | −4.89 | 2.59 | −8.00 |
| HH2 | −1.21 | −2.08 | −1.91 |
| | −2.08 | −7.81 | −0.91 |
| | −1.91 | −0.91 | −8.49 |
| N | 3.84 | 0.66 | −0.17 |
| | 0.66 | 4.92 | −0.34 |
| | −0.17 | −0.34 | 3.56 |
| HA | −1.50 | −0.02 | −0.19 |
| | −0.02 | −1.03 | 1.31 |
| | −0.19 | 1.31 | −5.29 |
| HB2 | 4.42 | −0.22 | 1.08 |
| | −0.22 | −0.47 | 0.38 |
| | 1.08 | 0.38 | −0.11 |
| HZ3 | 0.29 | −1.79 | −1.84 |
| | −1.79 | −1.83 | 1.04 |
| | −1.84 | 1.04 | −1.83 |
| HN | 0.51 | 1.54 | −0.62 |
| | 1.54 | 0.76 | −0.67 |
| | −0.62 | −0.67 | −0.73 |

**Table S2**. Elements (in MHz) of the TrpH$^{\bullet+}$ hyperfine coupling tensors, in the flavin principal axis system, appropriate for the orientation of $Trp_C$H in the *Cl*Cry4a crystal structure, calculated (by Dr Ilya Kuprov, Department of Chemistry, University of Southampton (4)) using density functional theory in Gaussian-03 (5) at the UB3LYP/EPR-III level for the radical cation of tryptophan.

| $[FAD^{\bullet-}\ Trp_CH^{\bullet+}]$ Dipolar Coupling Tensor / MHz | | |
|---|---|---|
| 1.09 | –12.12 | 4.91 |
| –12.12 | –7.80 | 7.02 |
| 4.91 | 7.02 | 6.71 |

**Table S3.** Elements (in MHz) of the dipolar coupling tensor, in the flavin principal axis system, appropriate for $[FAD^{\bullet-}\ Trp_CH^{\bullet+}]$ in the *Cl*Cry4a crystal structure.

A summary of the hyperfine tensors used in the various radical pair models in this work is given in Table S4.

| Radical pair model | FAD hyperfine tensors | TrpH hyperfine tensors |
|---|---|---|
| Fig. 2 | N5, N10, H1′, H1′′, 3 × H8 methyl protons | NE1, HE1, HB1, HE3 |
| Fig. 3 | All 15 | All 12 |
| Fig. S2, Fig. S4 | N5, N10, one H8 methyl proton | NE1, HE1, HB1, HE3 |
| Fig. S3 | N5, N10, H1′, H1′′, H6, 3 × H8 methyl protons and 2 of the 3 H7 methyl protons | None |

**Table S4**. Hyperfine tensors used in the various radical pair models in this work, indexed by figure number.

## S1.3. Direction of the radiofrequency field

The action-spectrum histograms presented in the main text were calculated using Eq. (1), in which the term $\left|\langle i|\hat{H}_{\perp}|j\rangle\right|^2$ was assumed to be proportional to the probability that a transition between two eigenstates of $\hat{H}_0$, $|i\rangle$ and $|j\rangle$, is induced by an RF field of frequency $\nu_{ij} = \left|\langle i|\hat{H}_0|i\rangle - \langle j|\hat{H}_0|j\rangle\right| / 2\pi$. The form of $\hat{H}_{\perp}$ in Eq. (1) assumes that the RF field is always perpendicular to the geomagnetic field. To check this assumption, we calculated an average histogram for [$FAD^{\bullet-}$ $TrpH^{\bullet+}$] using a truncated spin system (Table S4) with no dipolar coupling and randomly oriented RF fields. Histograms for each of the 199 randomly oriented geomagnetic field directions (Section S1.2) were averaged over 200 randomly oriented RF field directions. The result is shown in Fig. S2B along with the corresponding histogram for perpendicular geomagnetic and RF fields (Fig. S2A). The absolute difference in bar heights between the two is given in Fig. S2C, while Fig. S2D shows the root-mean-square deviation of the 39,800 histograms calculated for Fig. S2B from the histogram in Fig. S2A. Figs S2A and S2B are strikingly similar. The bar heights in Fig. S2C sum to ~0.1, i.e. around 10% of the total effect. The use of Eq. (1) is therefore sufficient to get a qualitative picture of the RF effects, especially given that the histograms increasingly resemble a uniform distribution up to the (effective) cut-off frequency as the radical pair model is refined by adding the dipolar interaction and a more realistic number of hyperfine-coupled nuclei.

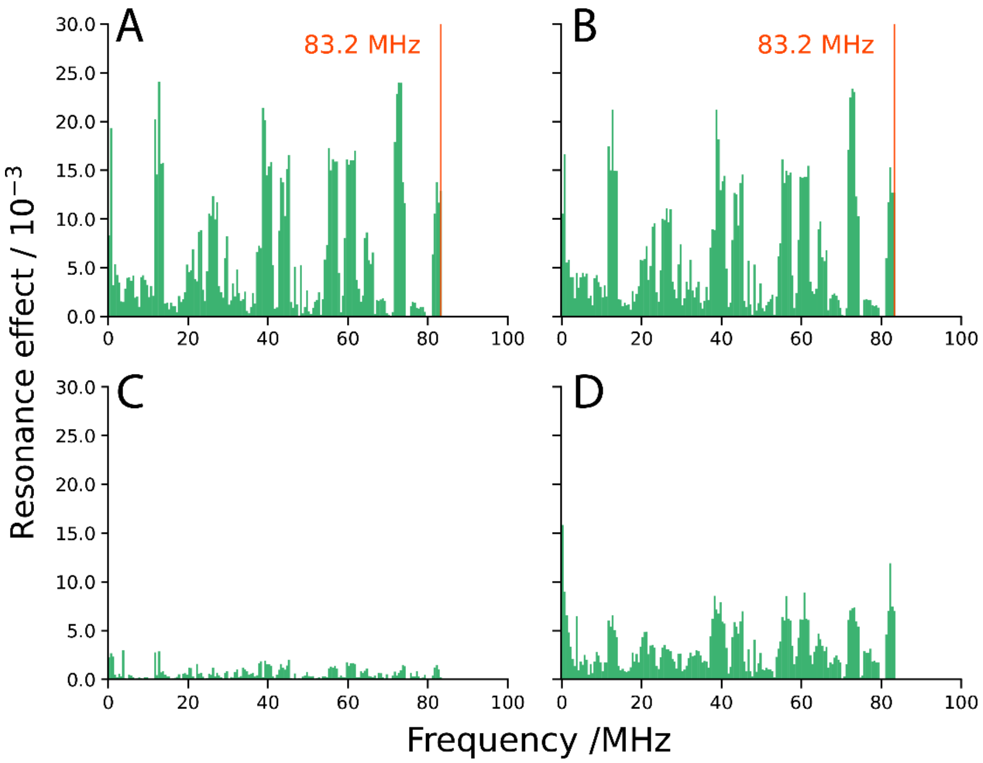


**Fig. S2**. Action-spectrum histograms. (A) Average histogram with the RF field perpendicular to the geomagnetic field, for 199 randomly oriented geomagnetic field directions. (B) Average histogram calculated with 200 RF randomly oriented field directions for each of the 199 geomagnetic field directions. (C) Absolute difference between the two average histograms in (A) and (B). (D) Root-mean-square deviation of the 39,800 histograms used to create (B) to the histogram in (A).

## S2. Supplementary Results: [$FAD^{\bullet-}$ $Z^{\bullet}$] radical pairs

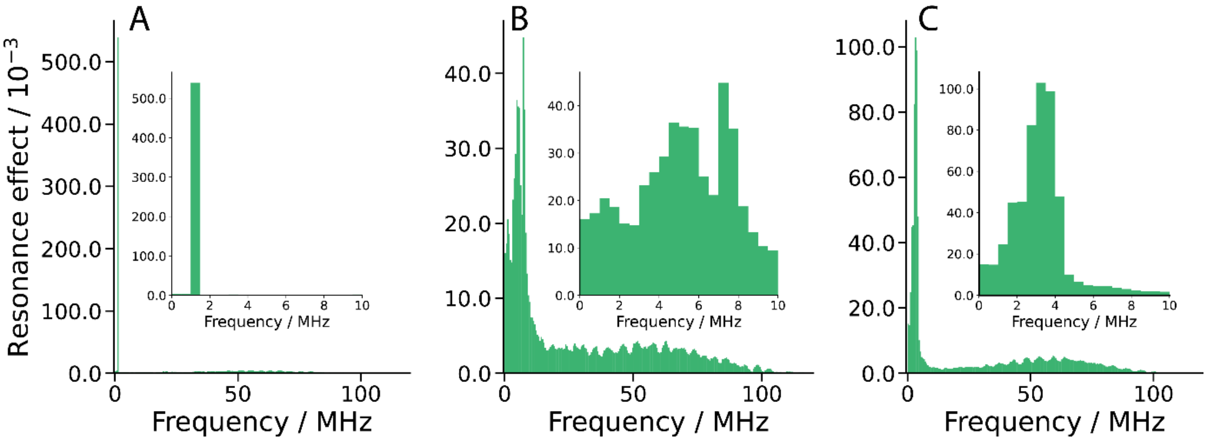


**Fig. S3**: Action spectrum histograms for [$FAD^{\bullet-}$ $Z^{\bullet}$], in the (A) absence and (B-C) presence of electron-electron dipolar coupling. The dipolar coupling strengths in (B) and (C) are $D$ = –14.3 MHz and $D$ = –8.1 MHz respectively. The insets in each panel are magnified versions of the graph for frequencies between 0 – 10 MHz.

Fig. S3A shows the action-spectrum histogram of a model [$FAD^{\bullet-}$ $Z^{\bullet}$] radical pair in which the $FAD^{\bullet-}$ radical contains 10 nuclear spins (Table S4) and $Z^{\bullet}$ is a hypothetical radical with no hyperfine interactions. In the absence of electron-electron coupling, $Z^{\bullet}$ behaves like an isolated electron, with electronic transitions occurring exclusively at the Larmor frequency of 1.4 MHz in a 50 μT geomagnetic field. This resonance in Fig. S3A completely dominates those arising from the $FAD^{\bullet-}$ radical.

Complete absence of electron-electron couplings, however, is unlikely. It is generally assumed that in a functional cryptochrome-based radical pair sensor, the rate of singlet recombination must be comparable to or preferably faster than the rate of electron spin relaxation. Sufficiently fast back electron transfer can only occur if the radicals are less than about 2 nm apart which means that their dipolar coupling is at least an order of magnitude bigger than the Earth's magnetic field and therefore far from negligible.

In contrast to Fig. S3A, the action-spectrum histogram for [$FAD^{\bullet-}$ $Z^{\bullet}$] including a dipolar interaction should bear greater resemblance to Figs S4B or S4C. We have arbitrarily chosen to use dipolar coupling tensors appropriate for [$FAD^{\bullet-}$ $TrpH^{\bullet+}$] radical pairs in cryptochrome, but any realistic dipolar coupling could have been used. The dipolar coupling tensors used for Figs S3B and S3C were appropriate for [$FAD^{\bullet-}$ $Trp_CH^{\bullet+}$] and [$FAD^{\bullet-}$ $Trp_DH^{\bullet+}$] in *Cl*Cry4a, respectively, with strengths $D$ = –14.3 MHz and $D$ = –8.1 MHz. The tensor for [$FAD^{\bullet-}$ $Trp_CH^{\bullet+}$], given in Table S3, was used for several of the other calculations presented here. The dipolar tensor for [$FAD^{\bullet-}$ $Trp_DH^{\bullet+}$] is given in Table S5 and its sole involvement in this work was its use to construct the model used for Fig. S3C.

| $[FAD^{\bullet-}\ Trp_DH^{\bullet+}]$ Dipolar Coupling Tensor / MHz | | |
|---|---|---|
| 2.42 | –6.15 | 1.41 |
| –6.15 | –7.18 | 2.89 |
| 1.41 | 2.89 | 4.76 |

**Table S5**. Elements (in MHz) of the dipolar coupling tensor, in the flavin principal axis system, appropriate for $[FAD^{\bullet-}\ Trp_DH^{\bullet+}]$ in the *Cl*Cry4a crystal structure.

The "coupled" $[FAD^{\bullet-}\ Z^{\bullet}]$ models contain the same 10 magnetic nuclei as the uncoupled model in Fig S3A. Resonances in the low-frequency region below 10 MHz (which is also magnified in the insets of each panel) are expected to have greater effect than regions of higher frequencies (> 10 MHz). However, the effect is clearly no longer dominated by Larmor-frequency transitions at 1.4 MHz.

The same conclusion, that a single frequency RF field at 1.4 MHz should not have a dominating effect on $[FAD^{\bullet-}\ Z^{\bullet}]$ radical pairs in the presence of a dipolar coupling, was also reached by Hiscock et al. (6), who also showed that broadband RF fields are expected to have greater disruptive effects on the magnetic compass sense. Figs 3(a) and 4(a) in Ref. (6) are the equivalents of Figs S3A and S3B, with differences being that only 7 nuclei were included in the model instead of 10, and a different dipolar coupling tensor ($D$ = –11.4 MHz) was used from the one in Table S3 ($D$ = –14.3 MHz).

The understanding gained from these calculations also has a bearing on the interpretation of behavioural tests that use ~1.4 MHz (Larmor frequency) RF fields (7-10). Disorientation effects at ~1.4 MHz and not, for example, at half or double that frequency, are only expected if two strict conditions are satisfied. First, one of the radicals must have all its hyperfine interactions smaller than 1.4 MHz. While this could be imagined (superoxide, $O_2^{\bullet-}$, for example, has been proposed as an alternative to $TrpH^{\bullet+}$ (8, 11) but see (4, 12, 13)), there is no independent evidence for the involvement of such a radical and little idea of its possible identity. Second, the dipolar coupling, $D$, should be less than 1.4 MHz, meaning that the radicals must be more than 3.8 nm apart (*SI Appendix*, Section S2). To put this in context, the third and fourth tryptophans of the Trp tetrad in *Er*Cry4a are 1.8 nm and 2.1 nm away from the centre of the flavin part of FAD. Even if a spin-correlated radical pair with a > 3.8 nm separation could be formed, its spin-selective recombination to the ground state (normally essential for a magnetic field effect) would be negligibly slow over such a large distance. This condition, and the problems associated with $O_2^{\bullet-}$ (12, 13) could be circumvented by Kattnig's radical scavenging mechanism (14-16). However, there is currently no experimental evidence for the existence of such a process in cryptochrome.

# S3. Supplementary Results: dipolar coupling as a perturbation

In the absence of electron-electron couplings, transitions involving both electron spins are strictly forbidden, and the cut-off frequency of the radical pair is the larger of the cut-off frequencies of the two radicals. In the case of [ $\mathrm{FAD}^{\bullet-}$ $\mathrm{TrpH}^{\bullet+}$ ], the cut-off frequency is that of $\mathrm{FAD}^{\bullet-}$.

Upon inclusion of electron-electron coupling, there are two main effects: (i) The frequency associated with each transition is shifted as a result of changes in the energies of the radical pair states. (ii) Transitions involving both electron spins become partially allowed. The consequences of (i) and (ii) can be seen by comparing Figs. 2C and 2D.

We first examine the consequences of (i) on the action-spectrum histogram. Shifts in the transition frequencies lead to the histogram bars being more “spread-out”, and an overall smoothing of the histogram is observed. Additionally, some transitions with frequencies slightly below the $\mathrm{FAD}^{\bullet-}$ cut-off are now shifted to a frequency above it. In Fig. 2, the dipolar coupling strength is $D = -14.3$ MHz, with fully allowed transitions shifted in this fashion appearing in the first ~10 MHz above the $\mathrm{FAD}^{\bullet-}$ cut-off.

We now consider effect (ii). Essentially, electron-electron coupling relaxes the strict rules forbidding transitions involving both electron spins. The stronger the electron-electron coupling, the more allowed such transitions become. To examine the originally forbidden transitions now “enabled” by the dipolar coupling, we constructed a model where the dipolar coupling is reduced in strength and treated as a perturbation on the uncoupled radical pair. The model contains seven hyperfine-coupled nuclei (see Table S4, section S1.2) and a dipolar coupling tensor equal to that in Table S3 multiplied by 0.01. For such a weak dipolar coupling, the transition frequencies are shifted by a maximum of 0.1 MHz. With an overwhelming majority of transitions remaining in the same histogram bin (bin width = 0.5 MHz), the effect (i) is largely negligible, allowing us to focus on (ii).

In the perturbation theory approach, the radical pair model is first constructed without the dipolar coupling. Then, we apply the weak dipolar coupling as a small perturbation, and make first-order corrections on the eigenvalues and eigenstates of the radical pair model. The advantage of this method is that we can keep track of whether each transition was previously allowed or forbidden in the absence of the dipolar coupling, and how allowed/forbidden it is after the perturbation. The transitions enabled by the dipolar coupling can hence be identified.

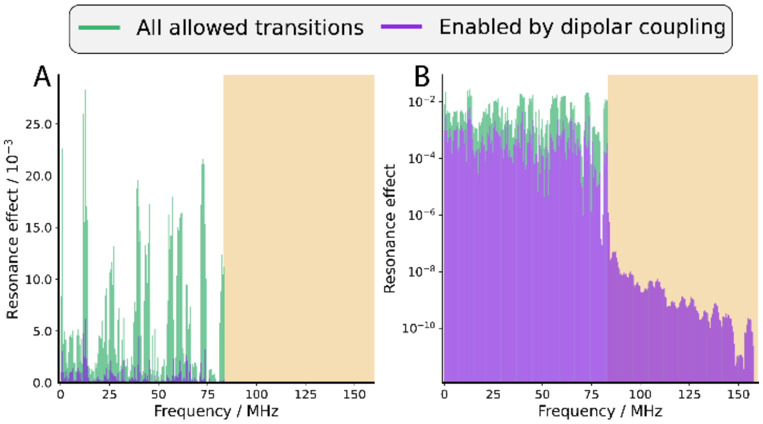


**Fig. S4**. Action-spectrum histogram of a simplified model of [ $FAD^{\bullet-}$ $TrpH^{\bullet+}$ ], on (A) a linear scale and (B) a logarithmic scale, where the dipolar coupling is 100 times smaller than its realistic value and applied as a perturbation of the uncoupled radical pair.

Fig. S4 shows the action-spectrum histogram of the model after the perturbation is applied. The full histogram is shown in green. The portion of the histogram that originates from transitions that are enabled by the dipolar coupling is plotted in purple. The region beyond the cut-off frequency of $FAD^{\bullet-}$ is shaded in pale orange, in which unsurprisingly all the transitions that appear are transitions enabled by the dipolar coupling.

The transitions newly enabled by the dipolar coupling account for only around 10% of the total effect, the overwhelming majority of which occur below the $FAD^{\bullet-}$ cut-off (white region). Fig. S4B shows that beyond the $FAD^{\bullet-}$ cut-off, the bar heights have magnitudes of around $10^{-6}$ to $10^{-10}$, their contributions to the total effect being negligible. By comparing Fig. S4B and the inset in Fig. 2D, we see that with the full dipolar coupling strength (100 times that used here), the bar heights beyond the $FAD^{\bullet-}$ cut-off (pale orange region) in Fig. 2D still only have magnitudes of $10^{-4}$ to $10^{-8}$, and hence still fail to account for a significant portion of the total effect.

# S4. Supplementary Methods: behavioural experiments

## S4.1. Testing site

The birds were kept in pairs (if possible) in on-site cages in a windowless room under a light regime imitating the local photoperiod, had access to food and water *ad libitum* and were surveyed by activity loggers to determine the onset of a switch to nocturnal activity. Behavioural experiments took place in specially constructed non-magnetic laboratory buildings described in earlier studies (17, 18) (full description in (19)), as soon as nocturnal activity could be detected in several birds. Within the laboratory, three aluminium-shielded chambers acting as Faraday cages allowed static magnetic fields to pass through, while attenuating time-dependent electromagnetic fields, ranging from 10 kHz to 10 GHz, by a factor of at least $10^5$. The electrical equipment used to generate the RF fields was grounded through an 8 m-deep earthing rod, while the individual chambers were grounded using single electrode loops in the laboratory base (19).

## S4.2. Generation and measurement of static magnetic field stimuli

Behavioural experiments were performed in two static magnetic field conditions, the normal magnetic field (NMF) and a changed magnetic field (CMF). In the NMF condition the local geomagnetic field of Oldenburg was present, while for the CMF case the horizontal component (declination) of the field was rotated 120° counter-clockwise, maintaining the local magnetic field strength and inclination angle. Static magnetic fields were generated by a double-wrapped, three-axis Merritt four-coil system in each of the three chambers (19-21). The rectangular coils measured ca. 2 m × 2 m. The behavioural experiments were conducted on a wooden table in the centre of the coil system where the homogeneity of the magnetic field was at least 99%. Each of the three sets of four coils was powered by a separate constant-current power supply (BOP 50–4 M, Kepco Inc., Flushing, NY, USA). In each chamber, the local and 120°-counter-clockwise rotated magnetic fields were recorded daily at alternating, opposite corners and in the centre of the experimental table using a flux-gate magnetometer (FVM-400, Meda Inc., Dulles, VA, USA).

The NMF condition was generated by running anti-parallel currents through the double-wrapped coils. Because the magnetic fields induced by currents running in opposite directions through the two windings in each coil exactly cancel one another, the birds perceived only the natural local geomagnetic field. In the CMF condition, the currents ran parallel through the double-wrapped coils, reinforcing instead of cancelling the fields they generated. The average magnetic measurements of the NMF over all test days and chambers were: autumn 2020 and 2021 (experimental series with 235-245 MHz fields): inclination 67.52° ± 0.004° (mean ± standard deviation (s.d.) for circular data) and intensity 48,845.28 ± 275.79 nT; spring 2022 (experimental series with 140-150 MHz fields): inclination 67.57° ± 0.003° (mean ± s.d.) and intensity 48,804.00 ± 258.35 nT. The average CMF measurements were: autumn 2020 and 2021: inclination 67.58° ± 0.005°, horizontal direction (declination) –120.07° ± 0.01° and intensity 48,827.76 ± 269.53 nT; spring2022: inclination 67.67° ± 0.01°, horizontal direction (declination) –120.08° ± 0.02° and intensity 48,755.42 ± 260.85 nT.

## S4.3. Generation and measurement of time-dependent electromagnetic fields

Every day, after the daily measurement of the static magnetic field, the magnetic components of the RF fields were measured in each chamber at opposite corners or edges of the tables. As recommended in Ref. (6) and carried out in Ref. (18), the fields were measured with a calibrated active loop antenna (Schwarzbeck Mess-Elektronik, HFS 1546, 150 kHz–400 MHz, Germany), placed 1.5 cm above the centre of the emission antennas. The measurement antenna was connected through the wall panel to a signal analyser (Rhode and Schwarz, FSV3004 Signal and Spectrum Analyser (10 Hz–4 GHz), Germany). The electric components of the fields were measured similarly with a calibrated active bi-conical antenna (Schwarzbeck Mess-Elektronik, EFS 9218 (9 kHz–300 MHz), Germany), connected to the signal analyser. The RF fields were recorded for 1 min daily with the resolution bandwidth of the analyser set to 10 kHz. Before and after a migratory

season, a one-hour measurement took place to record the RF fields applied during the experimental phase (magnetic component: Fig. S5; electric component: Fig. S6). For comparability with earlier studies (17-19, 22), we recorded two different spectral traces in each measurement: ‘maxhold’ (with the “Maxpeak” detector; for the detection of absolute maxima at a given frequency) and average spectral amplitudes (with the “RMS” detector; measuring the root-mean-square intensity over time at a given frequency). The spectral density (pT/√Hz) of the applied broadband noise signal in the frequency range of interest was integrated according to the equations in Ref. (17), see Table S8.

The RF fields were produced with one signal generator per experimental chamber (Rhode and Schwarz, SMBV100A (9 kHz–6 GHz) and SMBV100B (8 kHz–3 GHz), Germany), set to give broadband noise in the spectra range from 235 to 245 MHz (in autumn 2020 and 2021; centre frequency: 240 ± 5.75 MHz, resulting in a signal plateau width of 10 MHz; in the following with “-RF 240 MHz” suffix) and from 140-150 MHz (in spring 2022; centre frequency: 145 ± 5.75 MHz, also resulting in a signal plateau width of 10 MHz; in the following with “-RF 145 MHz” suffix). In the treatment condition (denoted with “RF” and the respective centre frequency in the following), the output RMS voltage at the signal generator was set to 37 mV, while in the control condition, the RMS voltage was set to 15 nV, so that in both conditions, the signal generators were actively producing a signal. Unless noted otherwise, coaxial cables (Schwarzbeck Mess-Elektronik, AK 9515 E, 50 Ohm, N-Connector, Germany) were used.

The signals were then fed into broadband power amplifiers (AR Deutschland GmbH, 50U1000 (10 kHz–1000 MHz), Germany), amplified to 60% of the maximum gain (ca. 45 dB) and guided into the experimental chambers via a wall panel. Inside the chambers, the signal was passed to a custom-built bandpass filter box (RF 240 MHz: attenuation up to 230 MHz; 3 dB attenuation at 230 MHz; RF 145 MHz: attenuation up to 71.88 MHz; 3 dB attenuation at 71.88 MHz), followed by an 8-Way splitter (Werlatone, Model D5829-10, 20–500 MHz, 400 W, N-Connectors, Patterson, NY, USA). Both were located under the experimental table within the Merritt-4 coil system. Each of the eight outputs from the splitter was fed into a coaxial cable (RG58C/U MIL-C-17, BNC connector), which was wrapped in a single turn around a custom-built, circular PVC antenna frame (diameter: 35.7 cm, height: 9 cm, circumference: 112.2 cm). The shield of the coaxial cable was removed along the circumference of the single turn coil, and the inner conductor was connected at one end to the shield at the other end to close the loop. This single-loop magnetic coil acted as an antenna, applying the generated broadband noise to the Emlen funnels placed inside. The generators were in ‘RF OFF’-mode and only switched to ‘RF ON’-mode for the experiments or during measurements.

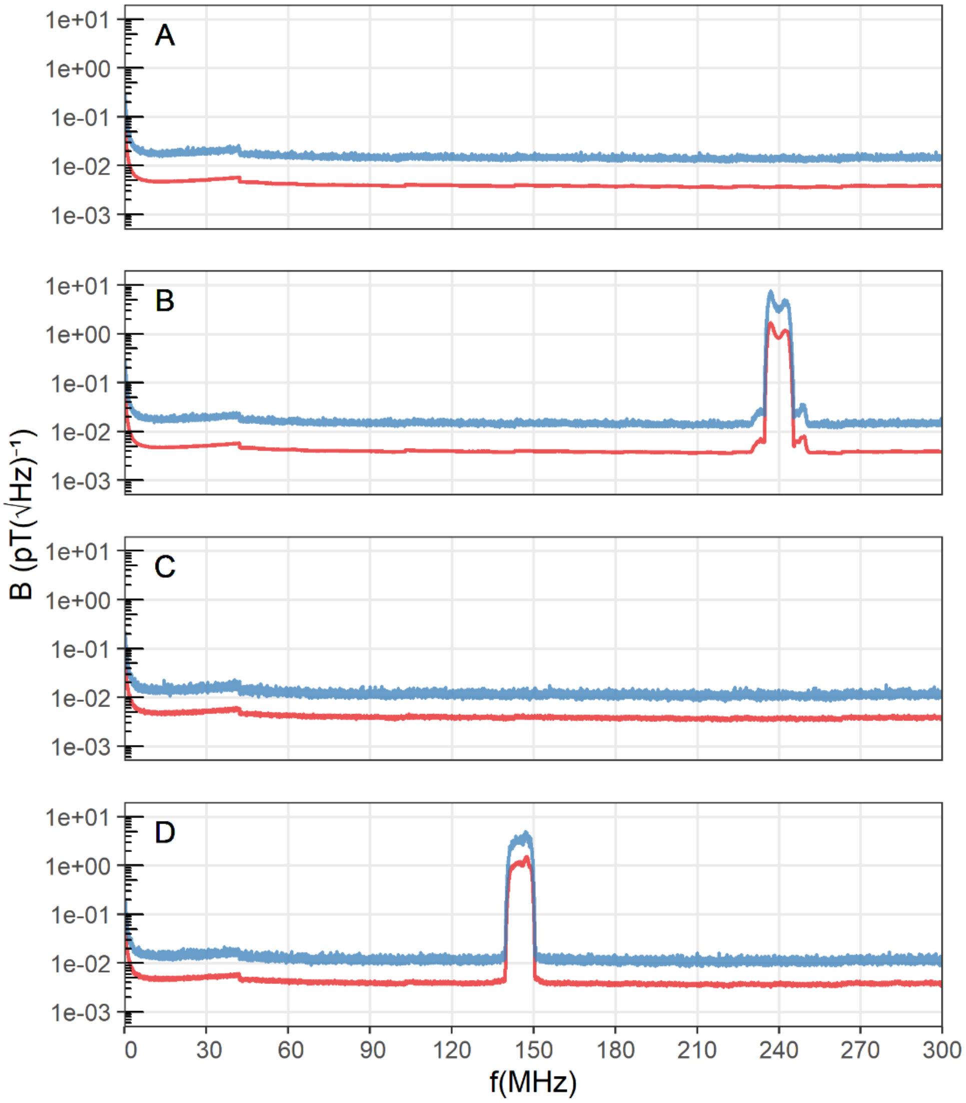


**Fig. S5**. Measurements of the magnetic components of the RF fields in the 150 kHz to 300 MHz range. (A) The control condition and (B) the 235-245 MHz broadband noise used for the "NMF-240" and "CMF-240" conditions in autumn of 2020 and 2021. (C) The control condition and (D) the 140-150 MHz broadband noise used for the "NMF-145" and "CMF-145" conditions in spring 2022. Spectral traces: 'average' (lower red line); 'maxhold' (upper blue line). Notice that the magnetic spectrum is extremely clean with no unwanted frequency peaks below the desired range.

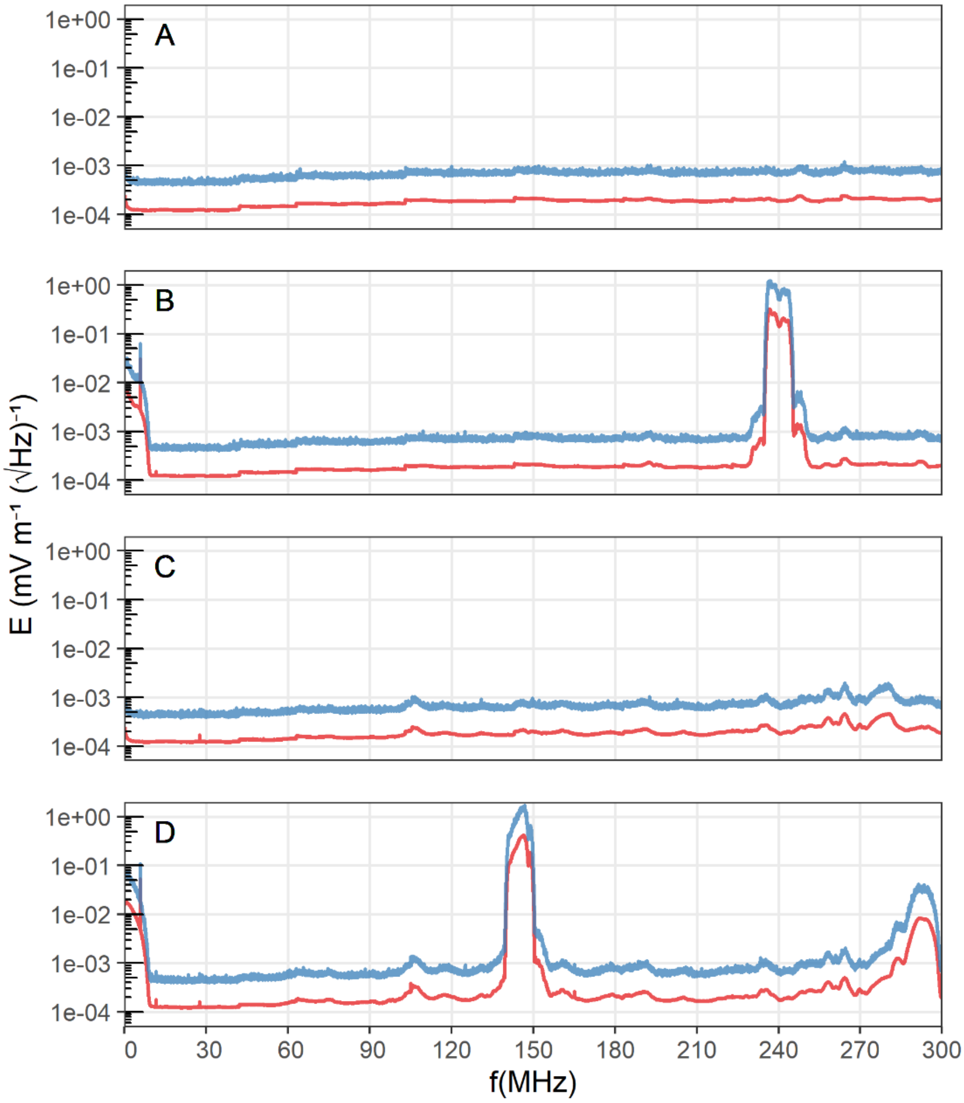


**Fig. S6**. Measurements of the electric components of the RF fields in the 150 kHz to 300 MHz range. (A) The control condition and (B) the 235-245 MHz broadband noise used for the "NMF-240" and "CMF-240" conditions in autumn of 2020 and 2021. (C) The control condition and (D) the 140-150 MHz broadband noise used for the "NMF-145" and "CMF-145" conditions in spring 2022. Spectral traces: 'average' (lower red line); 'maxhold' (upper blue line). The electric disturbance spectrum is as clean as that displayed in Fig. S5.

## S4.4. Acquisition and analysis of behavioural data

As soon as Eurasian blackcaps had been pre-screened by testing each individual's migratory motivation and ability to orient using their magnetic compass in the NMF and CMF conditions without RF-noise, they were tested in the control and RF-treatment conditions. The pre-screening results are not shown in Fig. 1, as these data were exclusively used for selection and excluded from further use. NMF and CMF data without RF-noise for the test series reported in Fig. 1 were collected from scratch after completing the pre-screening tests. On every test day, the birds were caught from the housing cages one hour before the end of civil twilight (approximately 30 min before sunset) to allow them to experience the sunset and potentially to calibrate their magnetic compass (23); but see Ref. (24). The birds were placed in the nearby botanical garden facing the sunset and sheltered from rain and wind. The boxes in which they were transferred to the nonmagnetic experimental laboratory were covered during transport to exclude any disturbance from streetlights. As in previous studies

(17-19, 25, 26), the behavioural tests were conducted under dim light (2.4 ± 0.04 mW $m^{-2}$), produced by incandescent light bulbs.

Behavioural experiments were performed using modified Emlen funnels made of white PVC (35 cm diameter, 15 cm high, walls 45° inclined) (27, 28). During the migratory season, night-migratory songbirds exhibit "migratory restlessness", expressed as jumping around and wing-whirring in a cage. During periods of migratory restlessness at night, the birds jump in the direction they want to migrate. The sloping sides of the funnels cause them to slide back down leaving marks on the scratch-sensitive thermal paper with which the funnels are lined, allowing the birds' intended migratory directions to be recorded. The edges of the scratch papers were joined with adhesive tape and the resulting overlaps were aligned with one of the cardinal directions, chosen at random for each experiment and measurement chamber.

In the experimental chambers, each bird was placed in a designated Emlen funnel lined with scratch-sensitive thermal paper. After one hour, the birds were returned to their transport boxes. Every test day consisted of two to three rounds of the same experimental condition with only the position of the funnel on the experimental table changed between rounds for each bird. After the last experimental round, the birds were returned to their housing cages. After every experimental round (lasting one hour), the scratch papers were collected, the funnels cleaned and, if applicable, prepared for another round. The behavioural tests were not blinded: the researcher who put the birds into the funnels knew the conditions under which they would be tested.

The scratch papers were analysed independently and in a blind manner by two researchers, unaware of the static magnetic field and RF field conditions, as well as the cardinal alignment of the scratch papers. Papers that had fewer than 30 scratches ("RF 240 MHz"-series in the autumns: 437 of 1207 papers, 36.2%; "RF 145 MHz"-series in the spring: 100 of 527 papers, 19.0%; previous studies (8, 9, 12, 16, 23): min-max: 21.6 – 40.0%) were classified as inactive and excluded from further analysis (7-9, 12, 15-16). For each of the remaining papers, the mean orientation of the scratches was determined to the nearest 10° and activity was scored (Score 1: 30-100 scratches, score 2: 100-300 scratches, score 3: 300-500 scratches, score 4: 500+ scratches; (29)). If the orientations estimated independently by the two researchers agreed to within 30°, the mean of the two values was taken as the bird's orientation ("RF 240 MHz"-series: 726 of 1207 papers, 60.1%; "RF 145 MHz"-series: 405 of 527 papers, 76.9%; previous studies (8, 9, 12, 16, 23): min-max: 53.0 – 69.9%). Otherwise, the paper was reassessed by a third evaluator and if the three directions still differed by more than 30° from either, the paper was deemed to be random ("RF 240 MHz"-series: 44 of 1207 papers, 3.6%; "RF 145 MHz"-series: 22 of 527 papers, 4.2%; previous studies (8, 9, 12, 16, 23): min-max: 6.0 – 11.7%). For every bird in each condition, its mean direction and directedness ($r$: mean resultant vector length) of all individual test results were calculated with a custom-written R-script (R Core Team 2013; "circular" package (30); "tidyverse" package (31)) using circular statistics. The data used for the final analysis is accessible in Table S9.

For each bird, the mean orientation of all tests in a given treatment condition was calculated. In line with previous studies (17, 18, 25), we included the means of all individuals with at least 3 directed tests in the relevant condition and $r \geq 0.2$ in Fig. 1. We also required that any given individual bird yielded a directional value in at least two of the four test conditions. For every experimental condition, the group mean orientation and directedness were then calculated by averaging over the mean direction of each individual bird. The group mean orientation was tested against the null hypothesis of a uniform circular distribution (Rayleigh test). The single experimental group with marginally significant orientation ($P = 0.12$) was compared with a directed experimental group using bootstrapping (18, 32-34). For the bootstrap, we merged the data from both years and resampled the data 100,000 times per condition randomly with replacement according to the sample size of the respective oriented condition in Fig. 1C.

We analysed these behavioural outcomes of the scratch paper evaluations (oriented, random, or inactive) in relation to the test conditions and test round with a binomial generalized linear mixed-effects model (GLMER). For the analysis we summarised all collected scratch papers depending on whether they were evaluated as oriented, random or inactive. We could not find any statistically significant influence of the test condition on the proportions of the behavioural outcome (across treatment series: at least $p > 0.07$; see Table S6), but that the individual bird always had a significant influence on the outcome of evaluation ($p < 0.0001$). While the test rounds had no effect on the proportion of papers evaluated as random (across treatment series: at least $p > 0.39$), they had a significant effect on the proportion of oriented and inactive papers ($p \leq 0.0009$ across treatment series), reflected in the proportion of oriented papers decreasing in the second round, while the proportion of inactive papers increased. We also analysed the activity scores of the active papers (categories ranging from 1 to 4; inactive papers were excluded) with a Skillings-Mack Test (a Friedman Test for data with missing values, since some birds were never scored with a certain category (35)). Once again, we could not find any significant effect of the test condition on the activity score (all four scores across treatment series: $p \geq 0.09$; see Table S7), but the test round turned out to influence the activity scores significantly in various of the categories across all migratory seasons ($p \leq 0.04$). These results suggest that the repeated tests per night have an effect on the birds' motivation to perform in the funnels. Since the individuals included in the final analysis still showed a considerably high directedness (*r*-value mean ± s.d.: "RF 240 MHz"-series: NMF = 0.47 ± 0.20; CMF = 0.39 ± 0.15; NMF-RF 240 MHz = 0.44 ± 0.22; CMF-RF 240 MHz = 0.46 ± 0.22; "RF 145 MHz"-series: NMF = 0.32 ± 0.11; CMF = 0.37 ± 0.13; NMF-RF 145 MHz = 0.38 ± 0.14; CMF-RF 145 MHz = 0.40 ± 0.16) we deem it unlikely that the orientations obtained varied too much between the rounds. Further, this has not been reported in previous funnel experiments with multiple rounds either (17-19, 22, 25, 26, 36). Considering that the birds were partially tested three rounds back-to-back (including handling) for several days a week, it seems conceivable that the individuals display some variability in their activity in the funnels across test rounds.

## S4.5. Elaboration on analysis of the autumn NMF control condition

In the bootstrap analysis of the group directedness (*r*-value) our analysis suggested that the oriented "NMF-240" lies well in the range of the bootstrapped *r*-values and vice versa (NMF: *r*: 0.3086, Confidence intervals (*CI*): 0.097 – 0.606, $p = 0.2618$; NMF-240: *r*: 0.437, *CI*: 0.233 – 0.675, $p = 0.9104$). To see whether the four test conditions of the "RF 240 MHz"-series differed in their spread, we compared the residuals from the respective mean in the NMF and CMF conditions, as well as the Control and "RF 240 MHz"-treatment conditions with each other. The applied Levene's Test ((37), applied in (38)), using the centre median for its robustness, did not yield any significant differences (NMF: $F_{(1, 40)} = 0.937$, $p = 0.3390$; CMF: $F_{(1, 42)} = 0.0008$, $p = 0.9780$; Controls: $F_{(1, 42)} = 0.875$, $p = 0.3548$; RF 240 MHz: $e_{(1, 40)} = 0.0001$, $p = 0.9913$). Observing that the spread around the mean did not differ between the conditions, we investigated how many individuals the non-significant condition would have required to reach a significant orientation, while retaining its *r*-value. This can be achieved by solving the formula to derive the *p*-value from the observed *r*-value and sample size for the sample size: $N = -\log(\alpha)/r^2$. Given an alpha level of 0.05 and the observed *r*-value of 0.3086, the NMF condition would have needed a sample size of 32. A visualization of this can be seen in Fig. S7A, where the directedness (*r*) is plotted over the sample size. The curve describing another reformulation of the above used formula makes the *r*-value act as a function of the sample size and the alpha level of 0.05: $r = \sqrt{-(2+4N+\log\alpha)\log\alpha} / 2N$ .

We next analysed whether our existing NMF data could possibly be significantly oriented while retaining its current sample size. Hence, we bootstrapped the 22 orientations from the autumn NMF experiment, drawing a randomised sample of 22 with replacement 100,000 times (seed = 30092022; note that these were newly generated, due to the different sample size compared to the bootstrap mentioned above). For each bootstrap sample, the *r*-value was computed. We then determined how many of the bootstrapped *r*-values were larger than the *r*-value of 0.369, required for significance for a sample size of

22. If less than 5% of the bootstrap samples exceed the significant threshold, the threshold should be considered unlikely given the value space of the original sample and thus should speak for non-directedness of the original sample. However, we found that more than 40% (43.1%) of the bootstrap samples have an *r*-value that exceeds the significance threshold of 0.369 (Fig. S7B), which therefore is by no means an extreme event. This suggests that the observed distribution in the autumn NMF would be well capable of surpassing the necessary directedness threshold for a significant orientation.

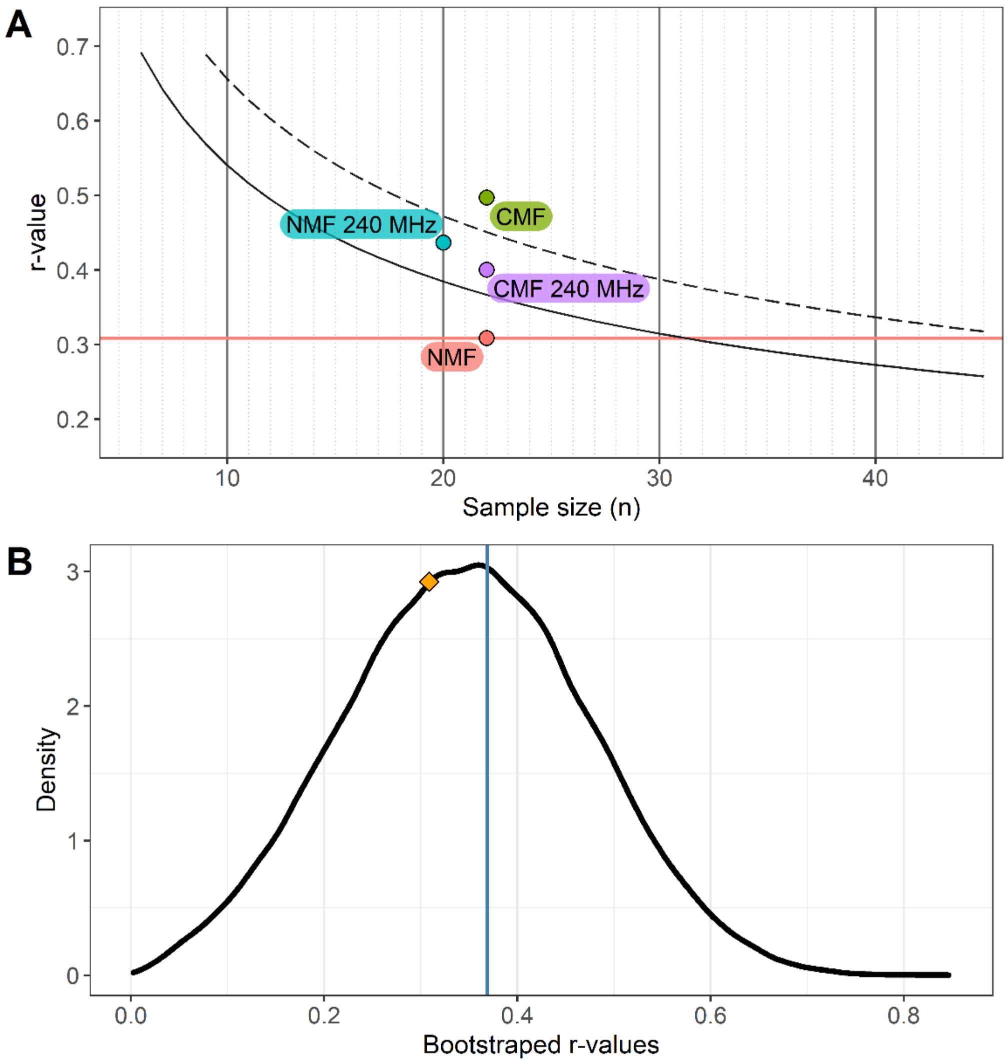


**Fig. S7**. (A) The relationship between the *r*-value over the sample size with a given significance threshold, plotted as a black line (solid: $\alpha = 0.05$; dashed: $\alpha = 0.01$), following the formula $r = \sqrt{-(2+4N+\log\alpha)\log\alpha} / 2N$ . The test conditions of the "RF 240 MHz"-series in autumn 2020 and 2021 are displayed as coloured circles and a coloured label at their respective sample size and *r*-value. Circles above the black lines correspond to test conditions with a significant group orientation. The red line represents the *r*-value of the NMF condition to highlight the required sample size, given the same directedness (*N* = 32). (B) Probability density distribution of the *r*-values from 100,000 bootstrap iterations drawn with replacement from the autumn NMF condition with its original sample size of 22. The observed *r*-value is marked with a diamond, and the corresponding significance threshold of 0.369 is indicated by a vertical blue line. 43.14 % of the bootstrapped samples have an *r*-value exceeding the significance threshold.

## S5. Supplementary Tables

| | term | Autumn 2020 + 2021<br>235-245 MHz<br>statistic | *d.f.* | *p*-value | Spring 2022<br>140-150 MHz<br>statistic | *d.f.* | *p*-value |
|---|---|---|---|---|---|---|---|
| Oriented | Individual | 128.77 | 1 | **< 0.0001** | 19.37 | 1 | **< 0.0001** |
| | Test condition | 4.35 | 3 | 0.2256 | 7.05 | 3 | 0.0705 |
| | Test round | 35.58 | 2 | **< 0.0001** | 16.82 | 2 | **0.0002** |
| Inactive | Individual | 210.82 | 1 | **< 0.0001** | 34.06 | 1 | **< 0.0001** |
| | Test condition | 5.560 | 3 | 0.1351 | 3.08 | 3 | 0.3790 |
| | Test round | 14.03 | 2 | **0.0009** | 22.55 | 2 | **< 0.0001** |
| Random | Individual | 168.77 | 1 | **< 0.0001** | 52.64 | 1 | **< 0.0001** |
| | Test condition | 4.11 | 3 | 0.2497 | 0.93 | 3 | 0.8186 |
| | Test round | 0.18 | 2 | 0.9150 | 1.89 | 2 | 0.3889 |

**Table S6**. Tabularised binomial GLM results of the analysis of the interaction between behavioural result (oriented, inactive, or random) test condition and test round, for the “RF 240 MHz”-series (middle) and the “RF 145 MHz”-series (right); listing the resulting test statistic, degrees of freedom (d.f.) and *p*-value of the respective interaction term. The analysis used all papers analysed in the respective migratory seasons, even if the bird was excluded from the final analysis, since only the behavioural outcome of the scratch paper is concerned here. A statically significant ($p \leq 0.05$) influence is indicated by a *p*-value in bold.

| | Activity | Autumn 2020 + 2021<br>235-245 MHz<br>*SM*-statistic | *d.f.* | *p*-value | Spring 2022<br>140-150 MHz<br>*SM*-statistic | *d.f.* | *p*-value |
|---|---|---|---|---|---|---|---|
| Test condition | Score 1 | 0.88 | 3 | 0.8308 | 3.96 | 3 | 0.2662 |
| | Score 2 | 2.86 | 3 | 0.4144 | 1.18 | 3 | 0.7575 |
| | Score 3 | 1.39 | 3 | 0.7081 | 1.74 | 3 | 0.6273 |
| | Score 4 | 2.01 | 3 | 0.5701 | 6.58 | 3 | 0.0865 |
| Test round | Score 1 | 29.13 | 2 | **< 0.0001** | 11.66 | 2 | **0.0029** |
| | Score 2 | 34.58 | 2 | **< 0.0001** | 0.66 | 2 | 0.7203 |
| | Score 3 | 17.85 | 2 | **0.0001** | 7.53 | 2 | **0.0232** |
| | Score 4 | 4.02 | 2 | 0.1341 | 6.28 | 2 | **0.0433** |

**Table S7**. Tabularised results of the Skillings-Mack tests performed on the independence of activity scores and test condition, as well as test round for the “RF 240 MHz”-series (middle) and the “RF 145 MHz”-series (right); reported are the resulting *Skillings-Mack*-statistic (*SM*-statistic), degrees of freedom (d.f.) and respective *p*-values. Like Table S6, all papers from the respective migratory seasons were used, even if the bird was excluded from the final analysis. Since only the activity of the bird on the scratch paper is concerned here, “inactive” papers were excluded. A statically significant ($p \leq 0.05$) influence is indicated by a *p*-value in bold.

| Frequency band (kHz) | Species | $\bar{b}^{\max}$ (pT/√Hz) | $\bar{b}$ (pT/√Hz) | $B_{\text{rms}}^{\max}$ (nT) | $B_{\text{rms}}$ (nT) | Orien-tation | Source |
|---|---|---|---|---|---|---|---|
| 10 – 5000 | ER | 0.07 | ~0.02 | 1.18 | ~0.37 | + | Ref. (22) (Fig. 4f, blue trace) |
| 10 – 5000 | ER | 5.58 | ~1.8 | 23.2 | ~7.35 | – | Ref. (22) (Fig. 4f, red trace) |
| 20 – 450 | ER | 30.1 | ~9.5 | 23.1 | ~7.29 | – | Ref. (22) (Fig. 4f, green trace) |
| 600 – 3000 | ER | 1.35 | ~0.43 | 2.3 | ~0.73 | – | Ref. (22) (Fig. 4f, black trace) |
| 0.1 – 100 | BC | 23.3 | 6.6 | 7.98 | 2.26 | + | Ref. (17) (Fig. 1a, RF-on) |
| 0.1 – 100 | BC | 0.92 | 0.32 | 1.29 | 0.51 | + | Ref. (17) (Fig. 1c, RF-off) |
| 75,000 – 85,000 | BC | 0.015 | 0.004 | 0.046 | 0.013 | + | Ref. (18) (Fig. 2A, Control) |
| 75,000 – 85,000 | BC | 2.525 | 0.701 | 8.681 | 2.404 | – | Ref. (18) (Fig. 2B, RF) |
| 235,000 – 245,000 | BC | 0.014 | 0.004 | 0.044 | 0.012 | + | Present study (Fig. S5A, RF-off) |
| 235,000 – 245,000 | BC | 3.601 | 0.961 | 12.359 | 3.286 | + | Present study (Fig. S5B, RF-on) |
| 140,000 – 150,000 | BC | 0.012 | 0.004 | 0.037 | 0.012 | + | Present study (Fig. S5C, RF-off) |
| 140,000 – 150,000 | BC | 2.813 | 0.948 | 9.463 | 3.181 | + | Present study (Fig. S5D, RF-on) |

**Table S8**. Comparative table of different noise density and intensity measures from (17, 18), with the results of the present study appended. Species: ER: European robin, BC: Eurasian blackcap. For formulas of $\bar{b}^{\max}$, $\bar{b}$, $B_{\text{rms}}^{\max}$, and $B_{\text{rms}}$, see (17) Eqs (2.1) and (2.3). Orientation: behavioural experiments resulted in oriented (+) or disoriented (–) birds.

**Table S9**. Experimental result summary for the individual birds in the respective test conditions separated by autumn and spring migratory season (NMF: normal magnetic field in Oldenburg; CMF: changed magnetic field, turned by 120° counter-clockwise; suffix "-RF 240 MHz": magnetic field condition with 235-245 MHz broadband RF fields present) ; suffix "-RF 145 MHz": magnetic field condition with 140-150 MHz broadband RF fields present). For each individual (Ring) the mean orientation (*dir*), and Rayleigh value (*r*) for this orientation, the portions of valid (valid), random (rnd) and not active (na) trials for the overall number of trials (*N*) are listed. Only result with sufficient valid trials and a directed orientation (valid ≥ 3; *r* ≥ 0.2) were used for the final orientation statistics and diagrams (Fig. 1 and 2 in the main article).

| | NMF | | | | | | CMF | | | | | | NMF-RF 240 MHz | | | | | | CMF-RF 240 MHz | | | | | | |
|---|---|---|---|---|---|---|---|---|---|---|---|---|---|---|---|---|---|---|---|---|---|---|---|---|---|
| Ring | dir | r | valid | rnd | na | N | dir | r | valid | rnd | na | N | dir | r | valid | rnd | na | N | dir | r | valid | rnd | na | N | Year |
| 02 yellow | 208.85 | 0.64 | 5 | 1 | 2 | 8 | 38.09 | 0.43 | 20 | 7 | 1 | 28 | 147.83 | 0.81 | 5 | 3 | 0 | 8 | 149.07 | 0.26 | 5 | 0 | 3 | 8 | 2020 |
| 19 violet | 152.10 | 0.51 | 7 | 1 | 6 | 14 | 128.99 | 0.54 | 6 | 0 | 19 | 25 | 194.45 | 0.78 | 6 | 0 | 4 | 10 | 358.27 | 0.17 | 7 | 0 | 24 | 31 | 2020 |
| 43 violet | 232.33 | 0.39 | 6 | 1 | 2 | 9 | 203.61 | 0.22 | 18 | 2 | 10 | 30 | 242.82 | 0.72 | 6 | 0 | 6 | 12 | 228.57 | 0.19 | 14 | 0 | 6 | 20 | 2020 |
| 45 blue | 121.77 | 0.66 | 6 | 2 | 8 | 16 | 64.80 | 0.37 | 4 | 0 | 4 | 8 | 219.29 | 0.24 | 5 | 0 | 6 | 11 | 51.30 | 0.23 | 8 | 0 | 12 | 20 | 2020 |
| 54 violet | 259.27 | 0.54 | 4 | 1 | 5 | 10 | 176.08 | 0.36 | 6 | 0 | 13 | 19 | 322.80 | 0.58 | 6 | 0 | 7 | 13 | 124.64 | 0.77 | 5 | 0 | 5 | 10 | 2020 |
| 62 orange/51 red | | | 0 | 0 | 6 | 6 | 210.00 | 1.00 | 1 | 0 | 1 | 2 | | | 0 | 0 | 2 | 2 | | | 0 | 0 | 6 | 6 | 2020 |
| 64 blue | 170.24 | 0.22 | 3 | 0 | 1 | 4 | 35.12 | 0.44 | 3 | 0 | 13 | 16 | 190.00 | 0.97 | 2 | 0 | 12 | 14 | 275.00 | 0.77 | 2 | 2 | 20 | 24 | 2020 |
| 71 violet | 173.23 | 0.86 | 6 | 0 | 4 | 10 | 132.84 | 0.57 | 5 | 0 | 9 | 14 | 147.41 | 0.70 | 9 | 3 | 3 | 15 | 54.91 | 0.20 | 8 | 1 | 7 | 16 | 2020 |
| 74 violet | 352.88 | 0.51 | 13 | 0 | 4 | 17 | 12.61 | 0.15 | 34 | 2 | 3 | 39 | 199.77 | 0.23 | 8 | 0 | 0 | 8 | 51.95 | 0.39 | 6 | 0 | 1 | 7 | 2020 |
| 75 violet | 286.38 | 0.20 | 7 | 0 | 4 | 11 | 178.89 | 0.57 | 5 | 1 | 8 | 14 | 24.62 | 0.19 | 7 | 0 | 5 | 12 | 152.36 | 0.50 | 4 | 1 | 14 | 19 | 2020 |
| 76 violet | 94.38 | 0.78 | 6 | 1 | 9 | 16 | 134.77 | 0.21 | 15 | 0 | 12 | 27 | 288.93 | 0.33 | 7 | 0 | 8 | 15 | 187.31 | 0.45 | 10 | 0 | 13 | 23 | 2020 |
| 88 yellow | 76.91 | 0.36 | 12 | 1 | 5 | 18 | 274.22 | 0.19 | 19 | 3 | 8 | 30 | 272.16 | 0.82 | 5 | 1 | 4 | 10 | 28.12 | 0.23 | 14 | 1 | 7 | 22 | 2020 |
| 100 orange | 323.73 | 0.12 | 29 | 0 | 12 | 41 | 123.56 | 0.52 | 4 | 0 | 6 | 10 | 233.09 | 0.35 | 7 | 1 | 4 | 12 | 100.75 | 0.29 | 5 | 0 | 1 | 6 | 2021 |
| 17 white | 320.10 | 0.27 | 22 | 0 | 16 | 38 | 343.02 | 0.39 | 11 | 0 | 3 | 14 | 347.67 | 0.25 | 13 | 0 | 12 | 25 | 137.50 | 0.23 | 4 | 0 | 4 | 8 | 2021 |
| 45 white | 285.22 | 0.34 | 3 | 0 | 6 | 9 | 61.97 | 0.33 | 5 | 0 | 7 | 12 | 65.40 | 0.18 | 12 | 0 | 17 | 29 | 182.84 | 0.70 | 5 | 0 | 13 | 18 | 2021 |
| 51 white | 174.45 | 0.78 | 7 | 0 | 2 | 9 | 4.32 | 0.08 | 16 | 0 | 2 | 18 | 261.27 | 0.35 | 5 | 1 | 2 | 8 | 146.47 | 0.51 | 5 | 0 | 3 | 8 | 2021 |
| 54 white | 21.01 | 0.01 | 25 | 0 | 17 | 42 | 55.57 | 0.21 | 14 | 0 | 2 | 16 | 13.26 | 0.25 | 17 | 0 | 4 | 21 | 85.14 | 0.82 | 4 | 0 | 0 | 4 | 2021 |
| 58 red | 263.25 | 0.36 | 5 | 0 | 0 | 5 | 81.96 | 0.62 | 6 | 1 | 1 | 8 | 290.37 | 0.24 | 5 | 2 | 1 | 8 | 165.19 | 0.90 | 6 | 0 | 0 | 6 | 2021 |
| 58 white | 40.00 | 1.00 | 1 | 0 | 4 | 5 | | | 0 | 0 | 10 | 10 | | | 0 | 0 | 6 | 6 | 335.00 | 1.00 | 1 | 0 | 7 | 8 | 2021 |
| 62 orange | 290.00 | 0.47 | 3 | 0 | 7 | 10 | 37.50 | 0.20 | 3 | 1 | 10 | 14 | 85.00 | 0.13 | 4 | 0 | 8 | 12 | 29.45 | 0.70 | 4 | 0 | 10 | 14 | 2021 |
| 67 violet | 279.31 | 0.38 | 9 | 0 | 6 | 15 | 164.42 | 0.37 | 7 | 0 | 7 | 14 | 197.93 | 0.59 | 5 | 0 | 5 | 10 | 150.08 | 0.54 | 6 | 1 | 9 | 16 | 2021 |
| 67 white | 285.46 | 0.32 | 8 | 0 | 5 | 13 | 56.73 | 0.34 | 7 | 0 | 1 | 8 | 315.59 | 0.15 | 16 | 0 | 6 | 22 | 284.56 | 0.12 | 14 | 0 | 4 | 18 | 2021 |
| 72 violet | 90.16 | 0.12 | 22 | 0 | 6 | 28 | 349.75 | 0.18 | 5 | 0 | 3 | 8 | 150.75 | 0.31 | 10 | 0 | 8 | 18 | 259.92 | 0.43 | 14 | 0 | 5 | 19 | 2021 |
| 72 white | 206.01 | 0.08 | 27 | 1 | 9 | 37 | 111.89 | 0.78 | 4 | 3 | 3 | 10 | 253.90 | 0.47 | 4 | 0 | 8 | 12 | 300.49 | 0.04 | 17 | 0 | 3 | 20 | 2021 |
| 74 white | 47.41 | 0.19 | 26 | 1 | 1 | 28 | 352.31 | 0.27 | 12 | 1 | 1 | 14 | 100.82 | 0.24 | 22 | 1 | 1 | 24 | 40.48 | 0.23 | 11 | 0 | 0 | 11 | 2021 |
| 81 violet | 245.22 | 0.67 | 5 | 0 | 4 | 9 | 165.42 | 0.33 | 6 | 0 | 4 | 10 | 205.43 | 0.15 | 14 | 0 | 10 | 24 | 244.99 | 0.44 | 20 | 0 | 4 | 24 | 2021 |
| 82 violet | 8.70 | 0.20 | 28 | 0 | 4 | 32 | 153.10 | 0.08 | 11 | 1 | 2 | 14 | 111.44 | 0.16 | 16 | 0 | 8 | 24 | 175.45 | 0.23 | 13 | 1 | 3 | 17 | 2021 |
| 88 violet | 213.36 | 0.57 | 9 | 1 | 0 | 10 | 191.46 | 0.32 | 13 | 1 | 0 | 14 | 203.07 | 0.37 | 6 | 0 | 0 | 6 | 233.05 | 0.63 | 22 | 0 | 0 | 22 | 2021 |
| 97 white | 2.65 | 0.32 | 28 | 1 | 2 | 31 | 133.55 | 0.27 | 4 | 0 | 2 | 6 | 304.38 | 0.24 | 7 | 0 | 1 | 8 | 350.18 | 0.39 | 9 | 0 | 1 | 10 | 2021 |

| | NMF | | | | | | CMF | | | | | | NMF-RF 145 MHz | | | | | | CMF-RF 145 MHz | | | | | | |
|---|---|---|---|---|---|---|---|---|---|---|---|---|---|---|---|---|---|---|---|---|---|---|---|---|---|
| Ring | dir | r | valid | rnd | na | N | dir | r | valid | rnd | na | N | dir | r | valid | rnd | na | N | dir | r | valid | rnd | na | N | Year |
| 58 red | 186.42 | 0.14 | 17 | 6 | 0 | 23 | 232.16 | 0.37 | 7 | 3 | 1 | 11 | 258.26 | 0.11 | 11 | 0 | 0 | 11 | 258.84 | 0.29 | 12 | 1 | 1 | 14 | 2022 |
| 72 violet | 25.57 | 0.32 | 6 | 0 | 5 | 11 | 300.43 | 0.21 | 3 | 0 | 5 | 8 | 50.32 | 0.25 | 7 | 1 | 9 | 17 | 75.82 | 0.19 | 9 | 0 | 8 | 17 | 2022 |
| 43 violet | 201.03 | 0.35 | 7 | 1 | 0 | 8 | 139.68 | 0.14 | 7 | 1 | 0 | 8 | 350.51 | 0.40 | 10 | 1 | 0 | 11 | 232.00 | 0.01 | 13 | 1 | 0 | 14 | 2022 |
| 67 white | 47.31 | 0.30 | 6 | 0 | 5 | 11 | 15.46 | 0.37 | 9 | 0 | 2 | 11 | 242.93 | 0.13 | 7 | 0 | 4 | 11 | 257.87 | 0.40 | 5 | 0 | 3 | 8 | 2022 |
| 97 orange | 82.39 | 0.64 | 10 | 1 | 0 | 11 | 218.51 | 0.29 | 8 | 0 | 0 | 8 | 153.56 | 0.20 | 14 | 2 | 1 | 17 | 181.04 | 0.09 | 15 | 1 | 1 | 17 | 2022 |
| 22 violet | 7.57 | 0.22 | 16 | 1 | 0 | 17 | 299.06 | 0.29 | 13 | 0 | 0 | 13 | 13.51 | 0.35 | 14 | 0 | 0 | 14 | 345.51 | 0.75 | 9 | 0 | 0 | 9 | 2022 |
| 44 white | 82.82 | 0.28 | 10 | 0 | 4 | 14 | 251.91 | 0.33 | 8 | 0 | 2 | 10 | 1.50 | 0.38 | 5 | 0 | 3 | 8 | 292.60 | 0.33 | 8 | 0 | 4 | 12 | 2022 |
| 45 white | 23.71 | 0.30 | 5 | 0 | 0 | 5 | 281.75 | 0.15 | 11 | 0 | 0 | 11 | 58.40 | 0.53 | 8 | 0 | 0 | 8 | 328.32 | 0.37 | 8 | 0 | 0 | 8 | 2022 |
| 82 violet | 162.07 | 0.35 | 10 | 2 | 2 | 14 | 302.87 | 0.46 | 4 | 0 | 3 | 7 | 14.56 | 0.48 | 8 | 0 | 3 | 11 | 340.77 | 0.07 | 8 | 1 | 5 | 14 | 2022 |
| 79 violet | 274.42 | 0.15 | 14 | 0 | 3 | 17 | 337.07 | 0.47 | 5 | 0 | 3 | 8 | 18.68 | 0.12 | 13 | 0 | 4 | 17 | 224.55 | 0.27 | 12 | 0 | 2 | 14 | 2022 |
| 88 violet | 294.60 | 0.18 | 11 | 0 | 0 | 11 | 243.07 | 0.72 | 7 | 0 | 1 | 8 | 111.98 | 0.32 | 7 | 0 | 1 | 8 | 241.58 | 0.66 | 8 | 1 | 0 | 9 | 2022 |
| 19 white | 345.46 | 0.25 | 14 | 2 | 1 | 17 | 0.57 | 0.05 | 8 | 2 | 0 | 10 | 69.50 | 0.64 | 7 | 0 | 1 | 8 | 226.28 | 0.43 | 10 | 5 | 0 | 15 | 2022 |
| 75 violet | 168.24 | 0.20 | 19 | 1 | 3 | 23 | 251.39 | 0.32 | 8 | 0 | 2 | 10 | 101.37 | 0.46 | 5 | 1 | 2 | 8 | 164.30 | 0.41 | 11 | 0 | 1 | 12 | 2022 |
| 99 orange | 5.21 | 0.37 | 6 | 0 | 5 | 11 | 201.73 | 0.42 | 7 | 0 | 6 | 13 | 76.10 | 0.21 | 11 | 0 | 3 | 14 | 332.71 | 0.30 | 7 | 0 | 5 | 12 | 2022 |
| 17 white | 85.06 | 0.22 | 7 | 0 | 3 | 10 | 276.29 | 0.23 | 6 | 0 | 2 | 8 | 15.79 | 0.19 | 16 | 0 | 1 | 17 | 355.03 | 0.39 | 7 | 0 | 1 | 8 | 2022 |
| 61 orange | 4.75 | 0.24 | 13 | 0 | 4 | 17 | 171.50 | 0.33 | 8 | 0 | 3 | 11 | 320.73 | 0.20 | 12 | 0 | 5 | 17 | 279.58 | 0.21 | 9 | 0 | 1 | 10 | 2022 |

## S6. Estimation of radiofrequency magnetic field effects on a magnetite-particle-based receptor

The magnetic particle hypothesis of magnetoreception assumes specialised neurons containing magnetic particles for the primary interaction with the Earth's magnetic field. The expected response of particles to a change in the magnetic field would be of mechanical nature (e.g., rotation (39)) and thus subject to viscous damping, which imposes a natural limit on the frequency at which mechanical motion could occur (between 100 Hz and 10 kHz, depending on the particle size (40)). Mechanical rotation of magnetic particles therefore is insignificant for magnetic field frequencies of 1 MHz and higher.

At these high frequencies, however, magnetic particles can absorb energy from the RF magnetic field by magnetic relaxation processes (e.g., (41)), which could potentially heat the tissue in which they occur. The heat production of magnetic particles in response to extremely weak RF magnetic fields (as used in magnetic orientation experiments) was found to be small but potentially not negligible by Shcherbakov & Winklhofer (42), who based their calculations on the then-used RF amplitude of 100 nT. However, the RF amplitudes that were found by Leberecht et al. (18) to produce disorientation at 80 MHz are significantly smaller (3.4 nT for $B_{\mathrm{rms}}$ = 2.4 nT), which is why we update the estimates in the following, again using the well-known formula for the loss power (e.g. (41)),

$$P = \frac{\pi \chi'' f B^2 n V}{\mu_0},$$

where $P$ is the dissipated power (in Watts), $\chi''$ is the dimensionless imaginary (dissipative) part of the magnetic susceptibility, $B$ is the RF magnetic field intensity (in Tesla), $f$ is the RF frequency and $n$ is the number of particles, each of which has volume $V$. Let us assume the magnetoreceptor contains $n$ = 10 magnetosome-like particles (magnetite cubes with 45 nm edge length) with $\chi''$ = 0.2 (43), then for $f$ = 80 MHz and $B$ = 3.4 nT, the total energy dissipated over 60 minutes RF exposure is as small as $0.4 k_{\mathrm{B}} T$ at physiological temperature and thus entirely negligible. To put this in perspective, in the magnetic hyperthermia technique, magnetic fields of typically 10 kA m$^{-1}$ (12.5 mT) at a driving frequency of 400 kHz (e.g. (44)) are commonly used to heat up tissue containing magnetic particles.

# S7. References


1. H. J. Hogben, M. Krzystyniak, G. T. P. Charnock, P. J. Hore, I. Kuprov, *Spinach* - A software library for simulation of spin dynamics in large spin systems. *J. Magn. Reson.* **208**, 179-194 (2011).
2. B. D. Zoltowski *et al.*, Chemical and structural analysis of a photoactive vertebrate cryptochrome from pigeon. *Proc. Natl. Acad. Sci. USA* **116**, 19449-19457 (2019).
3. J. C. S. Lau, C. T. Rodgers, P. J. Hore, Compass magnetoreception in birds arising from photo-induced radical pairs in rotationally disordered cryptochromes. *J. Roy. Soc. Interface* **9**, 3329-3337 (2012).
4. A. A. Lee *et al.*, Alternative radical pairs for cryptochrome-based magnetoreception. *J. R. Soc. Interface* **11**, 20131063 (2014).
5. M. J. Frisch *et al.* (2004) Gaussian 03. (Gaussian, Inc., Wallingford, CT).
6. H. G. Hiscock, H. Mouritsen, D. E. Manolopoulos, P. J. Hore, Disruption of magnetic compass orientation in migratory birds by radiofrequency electromagnetic fields. *Biophys. J.* **113**, 1475-1484 (2017).
7. P. Thalau, T. Ritz, K. Stapput, R. Wiltschko, W. Wiltschko, Magnetic compass orientation of migratory birds in the presence of a 1.315 MHz oscillating field. *Naturwissenschaften* **92**, 86-90 (2005).
8. T. Ritz *et al.*, Magnetic compass of birds is based on a molecule with optimal directional sensitivity. *Biophys. J.* **96**, 3451-3457 (2009).
9. K. Kavokin *et al.*, Magnetic orientation of garden warblers (*Sylvia borin*) under 1.4 MHz radiofrequency magnetic field. *J. R. Soc. Interface* **11**, 20140451 (2014).
10. R. Wiltschko *et al.*, Magnetoreception in birds: the effect of radio-frequency fields. *J. R. Soc. Interface* **12**, 20141103 (2015).
11. K. Maeda *et al.*, Magnetically sensitive light-induced reactions in cryptochrome are consistent with its proposed role as a magnetoreceptor. *Proc. Natl. Acad. Sci. USA* **109**, 4774-4779 (2012).
12. H. J. Hogben, O. Efimova, N. Wagner-Rundell, C. R. Timmel, P. J. Hore, Possible involvement of superoxide and dioxygen with cryptochrome in avian magnetoreception: origin of Zeeman resonances observed by *in vivo* EPR spectroscopy. *Chem. Phys. Lett.* **480**, 118-122 (2009).
13. T. C. Player, P. J. Hore, Viability of superoxide-containing radical pairs as magnetoreceptors. *J. Chem. Phys.* **151** (2019).
14. D. R. Kattnig, P. J. Hore, The sensitivity of a radical pair compass magnetoreceptor can be significantly amplified by radical scavengers. *Sci. Rep.* **7**, 11640 (2017).
15. D. R. Kattnig, Radical-pair-based magnetoreception amplified by radical scavenging: resilience to spin relaxation. *J. Phys. Chem. B* **121**, 10215-10227 (2017).
16. J. Deviers, F. Cailliez, A. d. l. Lande, D. R. Kattnig, Anisotropic magnetic field effects in the re-oxidation of cryptochrome in the presence of scavenger radicals. *J. Chem. Phys.* **156**, 025101 (2022).
17. D. Kobylkov *et al.*, Electromagnetic 0.1-100 kHz noise does not disrupt orientation in a night-migrating songbird implying a spin coherence lifetime of less than 10 microseconds. *J. R. Soc. Interface* **16**, 20190716 (2019).
18. B. Leberecht *et al.*, Broadband 75-85 MHz radiofrequency fields disrupt magnetic compass orientation in night-migratory songbirds consistent with a flavin-based radical pair magnetoreceptor. *J. Comp. Physiol. A* **208**, 97-106 (2022).
19. S. Schwarze *et al.*, Weak broadband electromagnetic fields are more disruptive to magnetic compass orientation in a night-migratory songbird (*Erithacus rubecula*) than strong narrow-band fields. *Front. Behav. Neurosci.* **10**, 55 (2016).
20. H. Mouritsen, "The Magnetic senses" in Neurosciences - from molecule to behavior: A university textbook, C. G. Galizia, P.-M. Lledo, Eds. (Springer-Verlag Berlin, 2013), pp. 427-443.
21. J. L. Kirschvink, Uniform magnetic fields and double-wrapped coil systems - improved techniques for the design of bioelectromagnetic experiments. *Bioelectromagnetics* **13**, 401-411 (1992).
22. S. Engels *et al.*, Anthropogenic electromagnetic noise disrupts magnetic compass orientation in a migratory bird. *Nature* **509**, 353-356 (2014).
23. W. W. Cochran, H. Mouritsen, M. Wikelski, Migrating songbirds recalibrate their magnetic compass daily from twilight cues. *Science* **304**, 405-408 (2004).
24. N. Chernetsov, D. Kishkinev, V. Kosarev, C. V. Bolshakov, Not all songbirds calibrate their magnetic compass from twilight cues: a telemetry study. *J. Exp. Biol.* **214**, 2540-2543 (2011).
25. N. Lefeldt, D. Dreyer, N. L. Schneider, F. Steenken, H. Mouritsen, Migratory blackcaps tested in Emlen funnels can orient at 85 degrees but not at 88 degrees magnetic inclination. *J. Exp. Biol.* **218**, 206-211 (2015).
26. S. Schwarze *et al.*, Migratory blackcaps can use their magnetic compass at 5 degrees inclination, but are completely random at 0 degrees inclination. *Sci. Rep.* **6**, 33805 (2016).
27. S. T. Emlen, J. T. Emlen, A technique for recording migratory orientation of captive birds. *Auk* **83**, 361-367 (1966).

28. H. Mouritsen, G. Feenders, A. Hegemann, M. Liedvogel, Thermal paper can replace typewriter correction paper in Emlen funnels. *J Ornithol* **150**, 713-715 (2009).
29. H. Mouritsen (1998) PhD thesis, Compasses and orientational strategies of night migrating passerines. (Odense University, Denmark).
30. C. Agostinelli, U. Lund, R package 'circular': Circular Statistics (version 0.4-93). https://r-forge.r-project.org/projects/circular/.
31. H. Wickham *et al.*, Welcome to the tidyverse. *J. Open Source Softw.* **4**, 1686 (2019).
32. N. I. Fisher, *Statistical analysis of circular data* (Cambridge University Press, Cambridge, 1995).
33. J. Bojarinova *et al.*, Magnetic compass of garden warblers is not affected by oscillating magnetic fields applied to their eyes. *Sci. Rep.* **10**, 3473 (2020).
34. B. Alert, A. Michalik, N. Thiele, M. Bottesch, H. Mouritsen, Re-calibration of the magnetic compass in hand-raised European robins (*Erithacus rubecula*). *Sci. Rep.* **5**, 14323 (2015).
35. M. Chatfield, A. Mander, The Skillings-Mack test (Friedman test when there are missing data). *Stata J* **9**, 299-305 (2009).
36. S. Engels, C. M. Hein, N. Lefeldt, H. Prior, H. Mouritsen, Night-migratory songbirds possess a magnetic compass in both eyes. *Plos One* **7**, e43271 (2012).
37. H. Levene, in Contributions to probability and statistics: essays in honor of Harold Hotelling I. Olkin, S. G. Ghurye, W. Hoeffding, W. G. Madow, H. B. Mann, Eds. (Stanford University Press, 1960), pp. 278–292
38. O. Lindecke, R. A. Holland, G. Petersons, C. C. Voigt, Corneal sensitivity is required for orientation in free-flying migratory bats. *Comm. Biol.* **4**, 522 (2021).
39. M. Winklhofer, J. L. Kirschvink, A quantitative assessment of torque-transducer models for magnetoreception. *J. Roy. Soc. Interface* **7**, S273-S289 (2010).
40. R. Kotitz, P. C. Fannin, L. Trahms, Time-domain study of Brownian and Neel relaxation in ferrofluids. *J. Magn. Magn. Mater.* **149**, 42-46 (1995).
41. R. Hergt, S. Dutz, M. Zeisberger, Validity limits of the Neel relaxation model of magnetic nanoparticles for hyperthermia. *Nanotechnology* **21**, 015706 (2010).
42. V. P. Shcherbakov, M. Winklhofer, Theoretical analysis of flux amplification by soft magnetic material in a putative biological magnetic-field receptor. *Phys Rev E* **81** (2010).
43. P. C. Fannin *et al.*, Investigation of the complex susceptibility of magnetic beads containing maghemite nanoparticles. *J. Magn. Magn. Mater.* **303**, 147-152 (2006).
44. R. Hergt *et al.*, Magnetic properties of bacterial magnetosomes as potential diagnostic and therapeutic tools. *J. Magn. Magn. Mater.* **293**, 80-86 (2005).
